# Effect of Micronutrients on Thyroid Parameters

**DOI:** 10.1155/2021/1865483

**Published:** 2021-09-28

**Authors:** Hari Krishnan Krishnamurthy, Swarnkumar Reddy, Vasanth Jayaraman, Karthik Krishna, Qi Song, Karenah E. Rajasekaran, Tianhao Wang, Kang Bei, John J. Rajasekaran

**Affiliations:** ^1^Vibrant Sciences LLC., San Carlos, CA, USA; ^2^Vibrant America LLC., San Carlos, CA, USA

## Abstract

Micronutrients are involved in various vital cellular metabolic processes including thyroid hormone metabolism. This study aimed to investigate the correlation between serum levels of micronutrients and their effects on thyroid parameters. The correlation of serum levels of micronutrients and thyroid markers was studied in a group of 387 healthy individuals tested for thyroid markers (T4, T3, FT4, FT3, TSH, anti-TPO, RT3, and anti-Tg) and their micronutrient profile at Vibrant America Clinical Laboratory. The subjects were rationalized into three groups (deficient, normal, or excess levels of micronutrients), and the levels of their thyroid markers were compared. According to our results, deficiency of vitamin B2, B12, B9 and Vit-D25[OH] (*p* < 0.05) significantly affected thyroid functioning. Other elemental micronutrients such as calcium, copper, choline, iron, and zinc (*p* < 0.05) have a significant correlation with serum levels of free T3. Amino acids asparagine (*r* = 0.1765, *p* < 0.001) and serine (*r* = 0.1186, *p* < 0.05) were found to have a strong positive correlation with TSH. Valine, leucine, and arginine (*p* < 0.05) also exhibited a significant positive correlation with serum levels of T4 and FT4. No other significant correlations were observed with other micronutrients. Our study suggests strong evidence for the association of the levels of micronutrients with thyroid markers with a special note on the effect of serum levels of certain amino acids.

## 1. Introduction

The deficiency of micronutrients such as vitamins and minerals is of great concern in public health. The World Health Organization (WHO) reported more than 2 billion people are affected by micronutrient deficiency and its related health consequences [1]. Iodine, iron, vitamin A, and zinc are the primary micronutrients that have been the focus of development efforts since they have major health implications. Micronutrient deficiency is regarded as a preventable cause of various nonspecific physiological impartments such as suppressed immune responses, metabolic disorders, and delayed or impaired physical and psychomotor development [2]. Elimination of micronutrient deficiencies through nutrition supplementation programs is widely seen as the most promising and cost-effective way to eradicate nutrition deficiency. The optimal metabolic functioning of an individual requires a proper supply of micronutrients such as vitamins, coenzymes, and intracellular elements. Micronutrients play a crucial role in catalyzing various enzymatic reactions, regulating the permeability of cell membranes, and various other physiological activities [3].

Nutritional alterations result in various endocrine dysfunctions with a prime effect on thyroid functioning. Thyroid disorders are the most common endocrine disorders and are known to affect 5% to 6% of the US population. Thyroid hormone is a sensitive hormone and synthesized by an autoregulated feedback loop mechanism regulated by the hypothalamus-pituitary-thyroid (HPT) axis. Thyroid hormones are involved in various developmental and physiological functioning. Regular functioning of a thyroid gland is characterized by the synthesis of the appropriate amount of triiodothyronine (T3) and thyroxine (T4) in response to thyroid-stimulating hormone (TSH) synthesized by the pituitary gland. Any physiological or biochemical alterations in the feedback loop result in thyroid dysfunctions and result in catastrophic health consequences. These alterations may arise from several pathologies; autoimmune disorders are the most common cause of thyroid disorders which results in excess (hyperthyroidism) or diminished (hypothyroidism) levels of thyroid hormones. Other reasons may include various environmental factors and demographic and intrinsic factors [4].

Autoimmune responses, thyroid surgery, radiation therapy, congenital hypothyroidism, etc. are the most commonly studied factors of thyroid dysfunctions. The pathogenesis of thyroid disorders has also been shown to be highly influenced by dietary factors, i.e., the availability of micronutrients such as iodine, vitamin D, iron, selenium, copper, zinc, vitamin B12, etc. Micronutrients are involved in physiological functioning like hormone synthesis, hormone transportation, and its binding to a target receptor. Micronutrients also play a pivotal role in regulating autoimmune thyroid disorders (AITD). Hypothyroidism is an autoimmune thyroid disorder resulting from iodine deficiency. The synthesis of both thyroid hormones triiodothyronine (T3) and thyroxine (T4) is inhibited by iodine deficiency which in turn induces the autoantibodies against the thyroid gland and results in goiter [3]. Hashimoto's thyroiditis (HT) is an autoimmune disorder characterized by hypothyroid functioning resulting from vitamin D deficiency. In addition to these nutrients, several other micronutrients such as amino acids, cofactors, and metal ions are essential for thyroid functioning. But only a few studies have reported the marginal possibilities of these micronutrients in altering thyroid functions. The present study is designed to evaluate the significant correlation between the serum levels of vital micronutrients and thyroid function.

## 2. Materials and Methods

### 2.1. Subjects and Study Design

The study population comprised 387 individuals aged between 13 and 85 subjects who were tested for various thyroid markers (thyroxine (T4), triiodothyronine (T3), free T4 hormone (FT4), thyroid-stimulating hormone (TSH), free triiodothyronine (FT3), antithyroid peroxidase (anti-TPO), reverse T3 (RT3), and antithyroglobulin (anti-Tg)) and micronutrient panel at Vibrant America Clinical Laboratory. The female to male ratio was 2 : 1 (69% female, 31% male), and the mean age (±SD) of the subjects was 48 ± 16 years. The study was exempted from formal ethical reviews by Western IRB (Washington, USA) since the study comprises the retrospective analysis of deidentified clinical data and test results. The subjects were categorized on the serum levels of thyroid markers listed in Table 1.

### 2.2. Reference Range of Thyroid Markers and Micronutrients

The reference ranges of thyroid markers and micronutrients tested depend on the lab where the test is performed. The present study followed the reference ranges widely followed by commercial diagnostic labs and hospital labs. The reference range of thyroid hormones and autoantibodies is shown in Table 1. The optimum serum levels of essential micronutrients are provided in Table S1.

### 2.3. Serum Analysis

Serum levels of TSH, FT4, anti-TPO, and anti-Tg tests were measured using a commercial Roche e601 analyzer (Roche Diagnostics, Indianapolis, IN, USA) following the manufacturer's instructions. All reagents were procured from Roche Diagnostics (Indianapolis, IN, USA).

Monoclonal antibodies specifically directed against human TSH were employed for the Elecsys TSH assay. The presence of chimeric construct from human- and mouse-specific components in antibodies labeled with ruthenium complex results in the elimination of interfering effects of HAMA (human anti-mouse antibodies).

For the Elecsys T4 and FT4 test, a specific anti-T4 antibody labeled with a ruthenium complex was used for the determination of free thyroxine. The use of a small quantity of the antibodies (equivalent to approx. 1-2% of the total T4 content of a normal serum sample) enables the equilibrium between bound and free T4 virtually unaffected. Serum levels of free triiodothyronine and bound triiodothyronine were determined using Elecsys FT3 assay, a specific anti-T3 antibody with a ruthenium complex.

Human antigen and monoclonal human anti-Tg antibodies were employed for the Elecsys anti-Tg assay whereas Elecsys anti-TPO assay used recombinant antigens and polyclonal anti-TPO antibodies for the determination of serum levels of anti-TPO.

Serum levels of RT3 were determined by a sensitive and reliable LC-MS/MS technique. Analytical standards of thyroid hormones were procured from Cerilliant Corporation (Round Rock, Texas), and the serum samples were analyzed using Waters TQ-S Tandem Mass Spectrometer. Serum levels of micronutrients were analyzed using Waters TQ-XS Tandem mass spectrometer (LC-MS MS), Waters GC-MS, and Perkin Elmer NexION ICP-MS using standard protocols.

### 2.4. Statistical Analysis

The processing of clinical data from deidentified subjects was performed via Java for windows version 1.8.161, and statistical analysis was performed using GraphPad Prism version 7.00 (Windows). Descriptive statistics were used to define continuous variables (mean ± SD, and median, minimum and maximum) with statistical significance set at *p* < 0.05. Mann–Whitney *U* test was used to compare two independent groups without normal distribution, and this method offers the advantage of possible comparison of small samples of subjects. Univariate relationships between variables were analyzed using Pearson's correlation analysis with significance set at *p* < 0.05.

## 3. Results

The present study aimed to evaluate the significance of 37 micronutrients on selected thyroid parameters. The study was conducted on the general population without any clinical prevalence of thyroid disorders. The mean age of the candidates involved in the study was 44 ± 14.5, and the study includes 70% females (Table 1).

The subjects were categorized into three groups based on the serum concentrations of micronutrients being less than the reference range, within the reference range, and higher than the reference. The analysis showed a significant relationship of selected micronutrients on the expression of thyroid hormones; decreased levels of amino acids such as asparagine, glutamine, serine, valine, citrulline, and arginine had a significant effect on thyroid parameters. Deficiency in these amino acids significantly alters the thyroid functioning, specifically, the deficiency of citrulline increased the serum levels of T4 (*p* < 0.001), and low levels of arginine decreased the serum levels of T3 (<0.0001). Thyroid functions were significantly affected by the various vitamin deficiencies; the present study observed that Vit B2, Vit B12, Vit B9(Folate), and Vit-D25[OH] are the most significant for normal thyroid functioning. Serum levels of T4 were significantly lower in subjects with low Vit B2 (*p* < 0.01). Vitamin B9 (folate) deficiency is the most significant factor affecting thyroid functioning as it increases the serum TSH level (*p* < 0.01), increases anti-TPO levels (*p* < 0.05), and also elevates the serum levels of anti-Tg (*p* < 0.001).

Apart from amino acids and vitamins, other micronutrients such as calcium, copper, chromium, selenium, inositol, and carnitine have significance on thyroid functioning. Decreased serum levels of carnitine were characterized by a significant increase in the levels of anti-TPO levels (*p* < 0.001) and anti-Tg (*p* < 0.01). While low levels of micronutrients are a concern, high levels can also result in adverse effects. Elevated levels of inositol and copper beyond the reference range have a significant increase in the serum levels of T4 and T3 (*p* < 0.001). Increased levels of selenium also show considerable significance in decreasing the levels of T3 and Free T3 (*p* < 0.01) (Table 2).

Pearson's correlation analysis between the micronutrient levels and thyroid parameters of the candidates elucidated the possible correlation between the micronutrients and thyroid function. The study observed that amino acids such as asparagine, serine, valine, leucine, and arginine have significant positive correlation with thyroid functioning. Asparagine (*r* = 0.1765, *p* < 0.001) and serine (*r* = 0.1186, *p* < 0.05) were found to have a strong positive correlation with serum levels of TSH. Amino acids valine and leucine exhibited a strong positive correlation with levels of T4 (*r* = 0.1474, *p* < 0.05) and free T4 (*r* = 0.1326, *p* < 0.05). Arginine was also found to have a significant positive correlation with T4 (*r* = 0.1592, *p* < 0.001). Micronutrients such as calcium, choline, copper, iron, and zinc have a significant strong positive correlation with T4 and free T4 levels (Table 3). Glutamine was found to have a negative correlation with T4 (*r* = −0.1955, *p* < 0.0001), and Vit E also exhibited a strong negative correlation with free T3 levels (*r* = −0.1594, *p* < 0.001). Other micronutrients such as coenzyme, cysteine, potassium, manganese, magnesium, chromium, and Vit A did not show a significant correlation with thyroid functioning.

## 4. Discussion

The present study details the association of essential micronutrients on thyroid functioning; thyroid dysfunction is one of the chronic disorders whose exact mechanism of pathogenesis remains unclear. Most of the thyroid disorders are related to subclinical derangements of thyroid functioning; abnormalities in thyroid functioning are usually screened based on the serum levels of thyroid parameters such as thyroxine (T4), triiodothyronine (T3), thyroid-stimulating hormone (TSH), and its related parameters or other investigations such as blood parameters, lipid profiling, cardiac dysfunctions, etc. Micronutrients, the most essential part of metabolic wellbeing, also have a vital role in thyroid health. Currently, evaluation of levels of micronutrients on thyroid functioning is limited to few nutrients such as iodine, selenium, vitamin D, and zinc [5]. The potential implication of other micronutrients was rarely considered in clinical investigation of thyroid dysfunctions; in our study, a total of 37 micronutrients including various vitamins, minerals, and amino acids were studied.

An extensive literature review suggested several studies on the association of micronutrients with thyroid parameters which are limited to few thyroid markers, but in the present study, the subjects were compared for 8 different thyroid parameters with a complete micronutrient profile. The study highlights the positive correlation of two nonessential amino acids asparagine and serine with serum levels of TSH. TSH is a glycoprotein hormone that contains unique asparagine-linked oligosaccharides on *α*-subunit. Baenziger and Green [6] have reported that the deficiency of serum levels of asparagine is linked with the inhibition of various proteins and also affects the synthesis of essential glycoproteins such as luteinizing hormone, follicle-stimulating hormone, and TSH. The asparagine-linked oligosaccharide glycoprotein TSH is also involved in the various cellular processes such as proper folding of endoplasmic reticulum and intracellular trafficking on Golgi apparatus [7]. Serine, a nonessential amino acid, naturally exists in its L-isomer form, and Mannisto et al. [8] have reported the ability of serine to cross the blood-brain barrier (BBB). This ability of serine enables it to reach the periventricular region and modulates the secretion of TSH. Reference [9] stated that low serum levels of thyroxine-binding globulin (serine protease inhibitor) are characterized by low levels of serum serine which increases the blood levels of free T4 by inhibiting the transportation of free T4 to the target tissue. Other amino acids such as valine, leucine, and arginine exhibited a strong positive correlation with serum levels of T4 and FT4. Numerous in vitro studies have demonstrated the importance of various amino acids in thyroid functioning. Mannisto et al. [8] reported the ability of glycine, glutamate, and serine to inhibit the TSH secretion by altering the functions of the periventricular hypothalamus and pituitary gland. This was evidenced by the ability of serine to diffuse through BBB. Tahara et al. [10] demonstrated the effects of amino acid deficiency on serum levels of T4, T3, free T4, and reverse T3; they reported that the reduction of phenylalanine and tyrosine drastically affects the serum levels of thyroid hormones. Tyrosine is an essential precursor molecule for thyroid hormone which is produced from another amino acid phenylalanine. Therefore, deficiency in any of these amino acids results in altered levels of thyroid hormones. There was no evidence for the participation of valine, leucine, and arginine in thyroid metabolism but their importance in immunological responses such as activation of T-cells, macrophages, and synthesis of various antibodies is well studied. Tahara et al. [10] reported that deficiency of any essential amino acids can directly alter T4 which results in primary hypothyroidism and various other thyroid disorders. This strongly supports the current findings where there is a positive correlation of essential amino acids valine, leucine, and arginine with serum levels of T4 and free T4.

Nonparametric analysis of subjects with vitamins deficiency and subjects with normal levels of vitamins by Mann–Whitney *U* test showed significant variation in serum levels of thyroid parameters. Anti-TPO levels were significantly high in subjects with folate deficiency; high levels of serum anti-TPO and anti-Tg are linked with the development of autoimmune hypothyroidism (Hashimoto thyroiditis). Hashimoto thyroiditis is characterized by T-cell mediated autoimmune response; serum concentrations of anti-TPO and anti-Tg vary the degree of thyroid hypofunction and cause intrathyroidal infiltration of B and T-cell CD4^+^ type 1 T helper cells. Several studies have reported the relationship between Vit-D25[OH] deficiency and elevated levels of anti-TPO; in this study, a significant relationship was observed between Vit-D25[OH] and serum levels of T3 and FT3 [11]. Although several studies including ours has implicated the role of vitamins in thyroid autoimmune disorders, the exact mechanism remains unclear due to diverse roles of vitamins [12]. The current study proposed that vitamins such as Vit-K, Vit-C, and Vit-B5 have no significant correlation with thyroid functioning.

Micronutrients such as zinc, copper, calcium, and iron exhibited a significant strong positive correlation with T4 and free T4. The prevalence of zinc in thyroid function is well known as its deficiency crucially depresses the serum levels of TSH, T4, and T3 by inhibiting TRH synthesis mediated in the pituitary gland. Zinc also plays a vital role in the conversion of inactive thyroid T4 to active thyroid T3 in the bloodstream [13]. Copper is another vital micronutrient that acts as a cofactor in various metabolic pathways. Copper deficiency inhibits the synthesis of both thyroid hormones by limiting the availability of tyrosinase activated by copper which synthesizes tyrosine, a protein component of thyroglobulin. Deficiency of both zinc and copper results in the decreased levels of thyroid hormones, hence resulting in hypothyroidism [14].

In the present study, it was observed that the micronutrients glutamine and vitamin A exhibited a negative correlation with thyroid functioning. Although the exact mechanism of the relation between Vit A and T3 was not clear, a report by Farhangi et al. [15] proved that the high levels of Vit A downregulate the TSH-*β* expression which in turn results in inhibition of TSH secretion and downregulation of thyroid hormone. The effect of glutamine on the thyroid mechanism remains unclear but Parry-Billings et al. [16] showed that individuals with hypo- or hyperthyroidism have decreased rates of glutamine released from the skeletal system.

One important limitation of our study which has to be considered while reviewing the data presented here is that the study includes free-living people of all ages and there was no collection of the diet records or lifestyle choices in the study population. To our knowledge, this is the first retrospective study on the association of a wide range of micronutrients and the incidence of total thyroid parameters. This study provides baseline data on the potential micronutrients involved in thyroid health.

## 5. Conclusion

The present study concludes that deficiency in essential micronutrients results in extreme derangement in thyroid functioning. The study also highlights the importance of serum levels of certain amino acids such as asparagine, serine, valine, leucine, and arginine are related to thyroid functioning. However, further detailed investigations are required to evaluate the physiological importance of these findings.

## Figures and Tables

**Table 1:** General characteristics of candidates involved in the study.

Category (*n* = 387)		Frequency	Percentage
Gender	Male	119	30.7
	Female	268	69.2

Thyroxine (T4) 0.40–4.50 mIU/mL	High	23	5.9
	Normal	361	93.2
	Low	3	0.78

Triiodothyronine (T3) 100–200 ng/dL	High	3	0.78
	Normal	375	96.9
	Low	9	2.3

Thyroid-stimulating hormone (TSH) 0.3–4.2 mlU/L	High	21	5.4
	Normal	360	93.0
	Low	6	1.55

Free thyroxine (FT4) 0.9–1.7 ng/dL	High	11	2.84
	Normal	373	96.3
	Low	3	0.78

Free triiodothyronine (FT3) 2.3–4.1 pg/mL	High	4	1.0
	Normal	381	98.4
	Low	2	0.52

Antithyroid peroxidase (anti-TPO) <9.0 IU/mL	High	44	11.3
	Normal	343	88.6
	Low	0	0

Reverse T3 (RT3) 9.2–24.1 ng/dL	High	27	6.98
	Normal	345	89.15
	Low	15	3.88

Antithyroglobulin (anti-Tg) <4.0 IU/mL	High	33	8.53
	Normal	354	91.47
	Low	0	0

**Table 2:** Micronutrients with significant association with thyroid parameters.

	Less than the reference range		Within the reference range		*P* ^a^ (*p* < 0.05)	Greater than the reference range		Within the reference range		*P* ^a^ (*P* < 0.05)
Vitamin B2 (5.6∼126.1 mcg/L)										
T4	6.7 ± 0.9	6.5 (4.9–8.6)	7.6 ± 1.6	7.4 (4.2–15.2)	0.0097	8.3 ± 1.3	8.3 (7.1–9.6)	7.6 ± 1.6	7.4 (4.2–15.2)	0.4948

Vitamin B12 (232∼1245 ng/L)										
FT4	1.6 ± 0.1	1.54 (1.5–1.6)	1.3 ± 0.2	1.3 (0.8–2.1)	0.0187	1.4 ± 0.2	1.29 (0.6–1.9)	1.3 ± 0.2	1.3 (0.8–2.1)	0.2569

Folate (≥1.5 ng/mL)										
TSH	5.7 ± 5.7	3.7 (1.4–19.9)	2.2 ± 1.5	1.8 (0.00–12.1)	0.0071					
ATPO	80.5 ± 101	20.4 (9.4–298.6)	24.8 ± 51.6	10.7 (5.01–556.1)	0.0125					
A-TG	104.8 ± 133.5	35.6 (14.6–406.3)	36.7 ± 74.1	13.47 (10–436.9)	0.0003					

Vitamin D25 (30.0∼108.0 ng/mL)										
T3	1.2 ± 0.3	1.1 (0.8–2.5)	1.1 ± 0.2	1.0 (0.5–3.0)	0.0006					
FT3	3.3 ± 0.4	3.1 (2.4–4.6)	3.1 ± 0.5	3.0 (2.3–3.9)	0.0076					

Selenium (109.8∼187.1 ng/mL)										
T3	1.1 ± 0.2	1.1 (0.7–1.5)	1.1 ± 0.2	1.1 (0.5–3)	0.8132	0.81 ± 0.2	0.8 (0.6–0.8)	1.1 ± 0.2	1.1 (0.5–3)	0.0032
FT3	3.1 ± 0.3	3.0 (2.5–4.1)	3.1 ± 0.4	3.1 (1.6–6.2)	0.7107	2.4 ± 0.12	2.55 (2.2–2.5)	3.1 ± 0.4	3.1 (1.6–6.2)	0.0014

Inositol (20.5∼51.4 nmol/mL)										
T4	7.9 ± 1.3	8.3 (4.6–9.7)	7.6 ± 1.6	7.4 (4.2–15.5)	0.2147	8.0 ± 1.2	7.57 (6.4–9.8)	7.6 ± 1.6	7.4 (4.2–15.5)	0.0009
T3	1.1 ± 0.2	1.07 (0.8–1.6)	1.1 ± 0.3	1.1 (0.5–3.0)	0.0535	1.2 ± 0.3	1.35 (0.6–1.5)	1.1 ± 0.3	1.1 (0.5–3.0)	0.0004

Carnitine (13.3∼39.6 nmol/ml)										
T3	1.4 ± 0.7	1.20 (0.7–3.0)	1.1 ± 0.2	1.11 (0.5–2.5)	0.0310	0.9 ± 0.2	1.0 (0.6–1.1)	1.1 ± 0.2	1.11 (0.5–2.5)	0.1660
ATPO	82.9 ± 72.4	51.2 (11.3 214.6)	24.9 ± 52.8	10.6 (5–556.1)	0.0002	10.2 ± 5	8.6 (5.4–18.3)	24.9 ± 52.8	10.6 (5–556.1)	0.3046
RT3	68.8 ± 148.3	12.8 (9–461)	14.3 ± 5.6	13.4 (4.8–39.6)	0.9782	32.4 ± 19.6	23 (17.6–65.8)	14.3 ± 5.6	13.4 (4.8–39.6)	0.0029
A-TG	94.8 ± 129	32.2 (11.1–419.8)	37 ± 74.8	13.5 (10–436)	0.0050	13.1 ± 2.2	12 (11.3–16.9)	37 ± 74.8	13.5 (10–436)	0.3369

Calcium (8.9∼10.6 mg/dL)										
FT3	2.9 ± 0.2	2.92 (2.6–3.3)	3.2 ± 0.5	3.1 (1.6–6.2)	0.0266	2.8 ± 0.3	2.7 (2.4–3.1)	3.2 ± 0.5	3.1 (1.6–6.2)	0.0667

Copper (0.7∼1.5 mcg/mL)										
T4	7.5 ± 1.3	7.2 (5.7–10.2)	7.5 ± 1.5	7.4 (4.2–15.5)	0.9109	10.3 ± 1.9	10.5 (7.3–14.2)	7.5 ± 1.5	7.4 (4.2–15.5)	<0.0001
T3	1.1 ± 0.1	1.06 (0.9–1.3)	1.1 ± 0.2	1.1 (0.5–3.0)	0.4881	1.5 ± 0.2	1.53 (1.1–1.8)	1.1 ± 0.2	1.1 (0.5–3.0)	<0.0001

Chromium (0∼0.7 ng/mL)										
FT4	1.2 ± 0.2	1.2 (0.9–1.6)	1.3 ± 0.2	1.3 (0.6–2.1)	0.0470	1.3 ± 0.1	1.3 (1.1–1.6)	1.3 ± 0.2	1.3 (0.6–2.1)	0.7680

Asparagine (30.0∼81.1 nmol/mL)										
FT3	3.1 ± 0.3	3.1 (2.1–3.7)	3.1 ± 0.4	3 (1.6–6.2)	0.0170	2.8 ± 0.2	2.8 (2.6–3.1)	3.1 ± 0.4	3 (1.6–6.2)	0.3781

Glutamine (278.0∼646.8 nmol/mL)										
T4	8.8 ± 2.4	7.8 (5.8–15.4)	7.5 ± 1.4	7.4 (4.2–15)	0.0232	7.1 ± 0.6	6.8 (6.5–8.1)	7.5 ± 1.4	7.4 (4.2–15)	0.5010

Serine (≥64.5 nmol/mL)										
T4	6.5 ± 1.1	6.5 (4.2–8.3)	7.6 ± 1.5	7.4 (4.3–15.4)	0.0250	7.8 ± 8.5	6.5 (4.2–8.3)	7.6 ± 1.5	7.4 (4.3–15.4)	0.5798
T3	0.9 ± 0.1	0.9 (0.6–1.26)	1.1 ± 0.2	1.1 (0.5–3.0)	0.0452	1.1 ± 1.4	1.8 (0.9–1.5)	1.1 ± 0.2	1.1 (0.5–3.0)	0.9471
Valine (136.0∼309.0 nmol/mL)										
T4	6.4 ± 1.2	6.7 (4.2–8.1)	7.6 ± 1.5	7.4 (4.4–15.5)	0.0156					
T3	1.0 ± 0.2	1.0 (0.5–1.3)	1.1 ± 0.3	1.1 (0.6–3.0)	0.02233					

Citrulline (17.0∼46.0 nmol/mL)										
T4	8.4 ± 1.5	7.7 (6.0–11.5)	7.6 ± 1.6	7.3 (4.2–15.5)	0.0066					
T3	1.2 ± 0.3	1.2 (0.8–1.8)	1.1 ± 0.2	1.1 (0.5–3.0)	0.0259					

Arginine (32.0∼120.0 nmol/mL)										
T4	7.1 ± 1.2	7.0 (4.2–9.8)	7.6 ± 1.6	7.4 (4.3–15.5)	0.0385					
T3	1.1 ± 0.3	1.0 (0.5–1.9)	3.1 ± 0.5	3.0 (1.8–6.2)	<0.0001					

Data are presented as the mean ± SD, median (min–max); ^a^Mann–Whitney *U* test.

**Table 3:** Micronutrients with significant Pearson's *r* correlation with thyroid parameters.

	*r*	*p*
Viatmin E		
FT3	−0.1594	0.0017
T3	−0.1036	0.0417

Vitamin B3		
A-TG	0.1235	0.015

Vitamin B6		
T4	−0.1161	0.0224
TSH	0.1424	0.005

Vitamin B12		
T3	−0.1142	0.0247

Vitamin D3		
T3	−0.1249	0.014
A-TG	0.1055	0.038

Vitamin K1		
T4	−0.1287	0.0113
FT4	−0.1781	0.0004

Vitamin D25 OH		
T4	−0.1027	0.0435
FT3	−0.1165	0.0218
T3	−0.1417	0.0052

Selenium		
FT4	0.115	0.0237

Carnitine		
T3	−0.1065	0.0363

Sodium		
ATPO	−0.1066	0.0361
RT3	−0.1341	0.0082

Calcium		
FT4	0.135	0.0078
FT3	0.1624	0.0013

Manganese		
FT4	−0.09603	0.0591

Zinc		
FT4	0.1511	0.0029
FT3	0.1195	0.0187

Copper		
T4	0.3462	<0.0001
T3	0.2789	<0.0001

Iron		
FT3	0.1981	<0.0001
TSH	−0.1263	0.0129

Isoleucine		
ATPO	−0.1101	0.0303

Valine		
T4	0.1474	0.0037
FT4	0.1327	0.009

Leucine		
T4	0.1474	0.0037
FT4	0.1326	0.009

Citrulline		
T4	−0.2274	<0.0001
FT4	−0.1089	0.0321
FT3	−0.1303	0.0103
T3	−0.1858	0.0002

Arginine		
T4	0.1592	0.0017

Asparagine		
TSH	0.1765	0.0005

Glutamine		
T4	−0.1955	0.0001
T3	−0.1005	0.0482
A-TG	−0.1363	0.0072

Serine		
TSH	0.1186	0.0196

Choline		
TSH	0.1621	0.0014

## Data Availability

The data used to support the findings of this study are available from the corresponding author upon request.

## Supplementary Materials

Supplementary Materials Table S1: detailed comparison of different thyroid parameters with micronutrients. Table S2: Pearson's *r* correlation of micronutrients with thyroid parameters.
