# Supplementary material for: Effect of Micronutrients on Thyroid Parameters
Source: J Thyroid Res. 2021 Sep 28;2021:1865483. doi: 10.1155/2021/1865483 (PMC8820928; doi:10.1155/2021/1865483)
Supplement: Supplementary Materials — Table S1: detailed comparison of different thyroid parameters with micronutrients. Table S2: Pearson's r correlation of micronutrients with thyroid parameters. [file 1865483.f1.docx]

**Table S1: Detailed comparison of different thyroid parameters with micronutrients**

ASPARAGINE (SERUM)

|  | Less than Reference  Range (n=37) | | Within range (n=368)  (30.0~81.1 nmol/mL) | | P  (P<0.05) | Greater than Reference  Range (n=02) | | Within range | | P  (P<0.05) |
| --- | --- | --- | --- | --- | --- | --- | --- | --- | --- | --- |
| T4 | 6.9 ± 0.9 | 6.8 (5.3-9.7) | 7.8 ± 0.8 | 7.4 (4.2-15.4) | 0.0655 | 7.6 ± 1.5 | 7.4 (4.2-15.4) | 7.8 ± 0.8 | 7.4 (4.2-15.4) | 0.7092 |
| T3 | 1.0 ± 0.1 | 1.1 (0.6-1.4) | 1.1 ± 0.25 | 1.1 (0.5-3.0) | 0.6805 | 1.2 ± 0.2 | 1.2 (1.0-1.5) | 1.1 ± 0.25 | 1.1 (0.5-3.0) | 0.3685 |
| TSH | 1.7 ± 0.8 | 1.5 (0.4-4.5) | 2.2 ± 1.5 | 1.8 (0.007-12) | 0.1372 | 10.5 ± 9.3 | 10.5 (1.2-19.9) | 2.2 ± 1.5 | 1.8 (0.007-12) | 0.6496 |
| FT4 | 1.2 ± 0.1 | 1.2 (1.1-1.5) | 1.3 ± 0.2 | 1.3 (0.6-2.1) | 0.2687 | 1.2 ± 0.1 | 1.2 (1.0-1.2) | 1.3 ± 0.2 | 1.3 (0.6-2.1) | 0.1559 |
| FT3 | 3.1 ± 0.3 | 3.1 (2.1-3.7) | 3.1 ± 0.4 | 3 (1.6-6.2) | 0.0170 | 2.8 ± 0.2 | 2.8 (2.6-3.1) | 3.1 ± 0.4 | 3 (1.6-6.2) | 0.3781 |
| ATPO | 20 ± 29.1 | 12.2(5.6-128) | 26 ± 54.6 | 10.7 (5-556) | 0.9546 | 45.9 ± 35.3 | 45.9 (10.6-81.3) | 26 ± 54.6 | 10.7 (5-556) | 0.3351 |
| RT3 | 12.4 ± 4.8 | 10.6 (5.6-24.8) | 15.7 ± 24 | 13.7 (4.8-460.9) | 0.0927 | 11.8 ± 1.4 | 11.8 (10.3-13.3) | 15.7 ± 24 | 13.7 (4.8-460.9) | 0.5417 |
| A-TG | 45.5 ± 96.6 | 14.4 (10.9-418.2) | 37.8 ± 75.4 | 13.5 (10-436.9) | 0.5434 | 14.9 ± 4.8 | 14.9(10.1-19.8) | 37.8 ± 75.4 | 13.5 (10-436.9) | 0.6601 |

GLUTAMINE (SERUM)

|  | Less than Reference  Range (n=24) | | Within range (n=359)  (278.0~646.8 nmol/mL) | | P  (P<0.05) | Greater than Reference  Range (n=4) | | Within range | | P  (P<0.05) |
| --- | --- | --- | --- | --- | --- | --- | --- | --- | --- | --- |
| T4 | 8.8 ±2.4 | 7.8 (5.8-15.4) | 7.5 ± 1.4 | 7.4 (4.2-15) | 0.0232 | 7.1 ± 0.6 | 6.8 (6.5-8.1) | 7.5 ± 1.4 | 7.4 (4.2-15) | 0.5010 |
| T3 | 1.2 ± 0.3 | 1.8 (0.8-2.4) | 1.1 ±0.2 | 1.1 (0.5-3) | 0.2865 | 0.9 ± 0.1 | 1 (0.7-1) | 1.1 ±0.2 | 1.1 (0.5-3) | 0.1614 |
| TSH | 1.9 ± 1.2 | 1.5 (0.3-5.1) | 2.2 ± 1.8 | 1.8 (0.007-19.9) | 0.2472 | 2.1 ± 1.3 | 1.5 (1.1-4.5) | 2.2 ± 1.8 | 1.8 (0.007-19.9) | 0.7124 |
| FT4 | 1.3 ± 0.2 | 1.3 (0.8-1.8) | 1.3 ± 0.2 | 1.2 (0.6-2.1) | 0.2282 | 1.3 ± 0.1 | 1.3 (1.1-1.5) | 1.3 ± 0.2 | 1.2 (0.6-2.1) | 0.7718 |
| FT3 | 3.1 ± 0.5 | 3.1 (2.3-4.5) | 3.1 ± 0. 4 | 3 (1.6-6.2) | 0.9107 | 2.7 ± 0.09 | 2.7 (2.5-2.8) | 3.1 ± 0. 4 | 3 (1.6-6.2) | 0.0155 |
| ATPO | 39.6 ± 72.1 | 12.1 (5.3-281) | 25 ± 52.3 | 10.7 (5.01-556) | 0.2909 | 12 ± 3.3 | 12.2 (7.5-16) | 25 ± 52.3 | 10.7 (5.01-556) | 0.8004 |
| RT3 | 15.6 ± 5.8 | 14.3 (8-30.5) | 15.5 ± 24.3 | 13.3 (4.8-460.97) | 0.2927 | 15 ± 3.11 | 15.2 (11.6-19.8) | 15.5 ± 24.3 | 13.3 (4.8-460.97) | 0.4203 |
| A-TG | 67.4 ± 107 | 13.9 (10.2-372.2) | 36.3 ± 73.8 | 13.5 (10-436) | 0.3285 | 15.5 ± 2.5 | 14.5 (13.3-19.7) | 36.3 ± 73.8 | 13.5 (10-436) | 0.4184 |

SERINE (SERUM)

|  | Less than Reference  Range (n=9) | | Within range (n=375)  (>=64.5 nmol/mL) | | P  (P<0.05) | Greater than Reference  Range (n=3) | | Within range | | P  (P<0.05) |
| --- | --- | --- | --- | --- | --- | --- | --- | --- | --- | --- |
| T4 | 6.5 ± 1.1 | 6.5 (4.2-8.3) | 7.6 ±1.5 | 7.4 (4.3-15.4) | 0.0250 | 7.8 ± 8.5 | 6.5 (4.2-8.3) | 7.6 ±1.5 | 7.4 (4.3-15.4) | 0.5798 |
| T3 | 0.9 ± 0.1 | 0.9 (0.6-1.26) | 1.1 ± 0.2 | 1.1 (0.5-3.0) | 0.0452 | 1.1 ± 1.4 | 1.8 (0.9-1.5) | 1.1 ± 0.2 | 1.1 (0.5-3.0) | 0.9471 |
| TSH | 1.6 ± 1.2 | 1.2 (0.4-4.5) | 2.2 ± 1.5 | 1.8 (0.007-12.05) | 0.1444 | 7.5 ±16.3 | 1.6 (1-19.9) | 2.2 ± 1.5 | 1.8 (0.007-12.05) | 0.9712 |
| FT4 | 1.3 ± 0.2 | 1.3 (0.8-1.8) | 1.3 ± 0.2 | 1.3 (0.6-2.1) | 0.7135 | 1.2 ± 1.4 | 1.03 (1-1.6) | 1.3 ± 0.2 | 1.3 (0.6-2.1) | 0.3884 |
| FT3 | 3.1 ± 0.5 | 3.1 (2.3-3.9) | 3.1 ± 0.4 | 3 (1.6-6.2) | 0.8466 | 2.8 ± 3.1 | 2.66 (2.6-3.2) | 3.1 ± 0.4 | 3 (1.6-6.2) | 0.2312 |
| ATPO | 72.5 ± 170 12.9 | 12.9 (8.7-556) | 24 ± 123 | 10.7 (5-324) | 0.3516 | 34.3 ± 67 | 11.4 (10.3-81.33) | 24 ± 123 | 10.7 (5-324) | 0.3765 |
| RT3 | 13.3 ± 4.3 | 13.7 (6.2-20.6) | 15.6 ± 23.8 | 13.4 (4.8-460) | 0.7813 | 12.4 ± 15.5 | 13.3 (8.1-15.6) | 15.6 ± 23.8 | 13.4 (4.8-460) | 0.6398 |
| A-TG | 26.2 ± 20 | 15.3 (10.6-79.4) | 43.9 ± 129 | 13.5 (10-436.9) | 0.1389 | 15.2 ± 18.4 | 13.2 (12.7-19.8) | 43.9 ± 129 | 13.5 (10-436.9) | 0.7901 |

COENZYME (SERUM)

|  | Less than Reference  Range (n=2) | | Within range (n=328)  (0.56~2.78 mcg/mL) | | P  (P<0.05) | Greater than Reference  Range (n=57) | | Within range | | P  (P<0.05) |
| --- | --- | --- | --- | --- | --- | --- | --- | --- | --- | --- |
| T4 | 7.7 ± 0.23 | 7.7 (7.5-8.0) | 7.4 ± 1.5 | 7.4 (4.2-15.4) | 0.6213 | 7.5 ± 1.5 | 7.5 (4.4-11.4) | 7.4 ± 1.5 | 7.4 (4.2-15.4) | 0.7398 |
| T3 | 1.0 ± 0.03 | 1.0 (1.04-1.1) | 1.1 ± 0.2 | 1.1 (0.5-3.02) | 0.7508 | 1.1 ± 0.2 | 1.1 (0.72-1.8) | 1.1 ± 0.2 | 1.1 (0.5-3.02) | 0.3335 |
| TSH | 1.5 ± 0.17 | 1.5 (1.3-1.7) | 2.2 ± 1.8 | 1.8 (0.007-19.9) | 0.5313 | 2.3 ± 1.6 | 1.9 (0.21-9.63) | 2.2 ± 1.8 | 1.8 (0.007-19.9) | 0.5207 |
| FT4 | 1.4 ± 0.24 | 1.46 (1.2-1.7) | 1.31 ± 0.21 | 1.29 (0.6-2.1) | 0.5312 | 1.3 ± 0.17 | 1.33(0.93-1.69) | 1.31 ± 0.21 | 1.29 (0.6-2.1) | 0.9188 |
| FT3 | 3.21 ± 0.07 | 3.21 (3.1-3.2) | 3.1 ± 0.49 | 3.1 (1.6-6.2) | 0.6454 | 3.0 ± 0.41 | 3.0 (2.21-4.52) | 3.1 ± 0.49 | 3.1 (1.6-6.2) | 0.2342 |
| ATPO | 8.8 ± 1.5 | 8.8 (7.3-10.3) | 27.5 ± 56.7 | 10.8 (5.0-556) | 0.3115 | 17.4 ± 30.6 | 9.96 (5.2-191) | 27.5 ± 56.7 | 10.8 (5.0-556) | 0.1335 |
| RT3 | 22.4 ± 2.6 | 22.4 (19.7-25) | 15.8 ± 25.3 | 13.5 (4.8-460) | 0.0451 | 14.0 ± 5.9 | 12.8 (5-36.2) | 15.8 ± 25.3 | 13.5 (4.8-460) | 0.5694 |
| A-TG | 13.8 ± 0.9 | 13.8 (12.9-14.8) | 37.6 ± 73.9 | 13.4 (1-436.9) | 0.9503 | 41.2 ± 90.2 | 14.05 (10-419) | 37.6 ± 73.9 | 13.4 (1-436.9) | 0.5883 |

SELENIUM (SERUM)

|  | Less than Reference  Range (n=22) | | Within range (n=362)  (109.8~187.1ng/mL) | | P  (P<0.05) | Greater than Reference  Range (n=3) | | Within range | | P  (P<0.05) |
| --- | --- | --- | --- | --- | --- | --- | --- | --- | --- | --- |
| T4 | 7.5 ± 1.2 | 7.5 (4.9-9.6) | 7.6 ± 1.5 | 7.4 (4.2-15.4) | 0.7603 | 6.6 ± 0.7 | 6.3 (5.9-7.7) | 7.6 ± 1.5 | 7.4 (4.2-15.4) | 0.2228 |
| T3 | 1.1 ± 0.2 | 1.1 (0.7-1.5) | 1.1 ± 0.2 | 1.1 (0.5-3) | 0.8132 | 0.81 ± 0.2 | 0.8 (0.6-0.8) | 1.1 ± 0.2 | 1.1 (0.5-3) | 0.0032 |
| TSH | 2.4 ± 1.3 | 2 (0.7-6.4) | 2.2 ± 1.8 | 1.8 (0.007-19.9) | 0.1299 | 2.2 ± 0.03 | 2.25 (2.2-2.28) | 2.2 ± 1.8 | 1.8 (0.007-19.9) | 0.3829 |
| FT4 | 1.2 ± 0.1 | 1.2 (0.9-1.6) | 1.3 ± 0.2 | 1.3 (0.6-2.14) | 0.4461 | 1.4 ± 0.13 | 1.4 (1.2-1.57) | 1.3 ± 0.2 | 1.3 (0.6-2.14) | 0.3136 |
| FT3 | 3.1 ± 0.3 | 3.0 (2.5-4.1) | 3.1 ± 0.4 | 3.1 (1.6-6.2) | 0.7107 | 2.4 ± 0.12 | 2.55 (2.2-2.5) | 3.1 ± 0.4 | 3.1 (1.6-6.2) | 0.0014 |
| ATPO | 30 ± 48.3 | 11.3 (5.3-155) | 25.8 ± 54.2 | 10.6 (5-556) | 0.7670 | 11.1 ± 3.3 | 13 (6.4-13.8) | 25.8 ± 54.2 | 10.6 (5-556) | 0.9327 |
| RT3 | 12.9 ± 4.9 | 12.4 (7-27.3) | 15.7 ± 24.2 | 13.6 (4.8-460.9) | 0.1987 | 15.6 ± 3.6 | 15.2 (11.3-20.3) | 15.7 ± 24.2 | 13.6 (4.8-460.9) | 0.5559 |
| A-TG | 29 ± 34.6 | 13.3 (10-136.7) | 38.7 ± 78.4 | 13.5 (10-436.9) | 0.8124 | 22.9 ± 16.8 | 11.9 (10.2-46.7) | 38.7 ± 78.4 | 13.5 (10-436.9) | 0.4869 |

ZINC (SERUM)

|  | Less than Reference  Range (n=10) | | Within range (n=370)  (0.5~0.9 mcg/mL) | | P  (P<0.05) | Greater than Reference  Range (n=7) | | Within range | | P  (P<0.05) |
| --- | --- | --- | --- | --- | --- | --- | --- | --- | --- | --- |
| T4 | 8 ± 1.2 | 7.4 (6.4-10.2) | 7.5 ± 1.5 | 7.4 (4.2-15.4) | 0.3216 | 8.0 ± 1.0 | 8.0 (6.7-9.5) | 7.5 ± 1.5 | 7.4 (4.2-15.4) | 0.2413 |
| T3 | 1.1 ± 0.3 | 1.1 (0.6-1.5) | 1.1 ± 0.2 | 1.1 (0.5-3.0) | 0.8682 | 1.0 ± 0.14 | 1.1 (0.9-1.2) | 1.1 ± 0.2 | 1.1 (0.5-3.0) | 0.7195 |
| TSH | 2.0 ± 1.1 | 1.5 (0.6-5.1) | 2.2 ± 1.7 | 1.8 (0.007-19.9) | 0.5761 | 2.5 ± 1.5 | 2.3 (0.4-5.6) | 2.2 ± 1.7 | 1.8 (0.007-19.9) | 0.4560 |
| FT4 | 1.3 ± 0.18 | 1.26 (1-1.82) | 1.3 ± 0.2 | 1.3 (0.6-2.1) | 0.6645 | 1.4 ± 0.19 | 1.34 (1.2-1.8) | 1.3 ± 0.2 | 1.3 (0.6-2.1) | 0.3561 |
| FT3 | 2.9 ± 0.4 | 3.0 (1.8-3.61) | 3.1 ± 0.4 | 3.0 (1.6-6.2) | 0.3419 | 3.2 ± 0.42 | 3.3 (2.5-3.9) | 3.1 ± 0.4 | 3.0 (1.6-6.2) | 0.5012 |
| ATPO | 26.7 ± 40 | 12.3 (5.3-147) | 26 ± 54.4 | 10.7 (5-556) | 0.5147 | 17.3 ± 13.9 | 14 (8-50.8) | 26 ± 54.4 | 10.7 (5-556) | 0.4990 |
| RT3 | 19.5 ± 15.7 | 13.6 (10.3-65.8) | 15.4 ± 23.8 | 13.4 (4.8-460.9) | 0.3875 | 14.9 ± 2.6 | 15.7 (11.2-18.2) | 15.4 ± 23.8 | 13.4 (4.8-460.9) | 0.3959 |
| A-TG | 19.7 ± 22.3 | 10.9 (10.1-86.3) | 38.9 ± 77.9 | 13.5 (10-436.9) | 0.0457 | 15 ± 3.0 | 14.4 (10.9-20) | 38.9 ± 77.9 | 13.5 (10-436.9) | .7767 |

CYSTEINE (SERUM)

|  | Less than Reference  Range (n=9) | | Within range (n=362)  4.4~28.9 nmol/mL | | P  (P<0.05) | Greater than Reference  Range (n=16) | | Within range | | P  (P<0.05) |
| --- | --- | --- | --- | --- | --- | --- | --- | --- | --- | --- |
| T4 | 7.4 ± 0.7 | 7.7 (6.6-8.2) | 7.6 ± 1.6 | 7.4 (4.2-15.2) | 0.9107 | 7.4 ± 1.3 | 7.4 (5.3-10) | 7.6 ± 1.6 | 7.4 (4.2-15.2) | 0.7312 |
| T3 | 1.0 ± 0.2 | 1.05 (0.5-1.2) | 1.1 ± 0.3 | 1.11 (0.6-3.0) | 0.1589 | 1.1 ± 0.2 | 1.07 (0.8-1.4) | 1.1 ± 0.3 | 1.11 (0.6-3.0) | 0.6064 |
| TSH | 1.9 ± 0.7 | 2.04 (0.6-2.7) | 2.2 ± 1.8 | 1.83(0.007-19.9) | 0.9809 | 2.7 ± 1.4 | 2.54 (0.9-5.9) | 2.2 ± 1.8 | 1.83(0.007-19.9) | 0.1223 |
| FT4 | 1.3 ± 0.2 | 1.3 (1.0-1.7) | 1.3 ± 0.2 | 1.3 (0.6-2.1) | 0.7705 | 1.3 ± 0.2 | 1.25 (1.0-1.7) | 1.3 ± 0.2 | 1.3 (0.6-2.1) | 0.4934 |
| FT3 | 2.9 ± 0.6 | 2.8 (1.6-4.1) | 3.2 ± 0.5 | 3.1 (1.8-6.2) | 0.0866 | 3.1 ± 0.5 | 3.0 (2.4-4.2) | 3.2 ± 0.5 | 3.1 (1.8-6.2) | 0.3686 |
| ATPO | 32 ± 28.1 | 12.7 (8.0-79.0) | 25.6 ± 53.0 | 10.7(5.0-556.1) | 0.1819 | 30.8 ± 76.0 | 10.7 (5.4-324.5) | 25.6 ± 53.0 | 10.7(5.0-556.1) | 0.7121 |
| RT3 | 17.2 ± 6.2 | 14.4 (9.7-28.4) | 15.5 ± 24.2 | 13.2 (4.8-461) | 0.1440 | 17.4 ± 5.7 | 16.4 (7.7-28.0) | 15.5 ± 24.2 | 13.2 (4.8-461) | 0.0200 |
| A-TG | 34.2 ± 44.6 | 14.8 (12.3-157.8) | 38.1 ± 77.6 | 13.5(10.0-436.9) | 0.1643 | 38.4 ± 61.1 | 13.3 (11.3-231.8) | 38.1 ± 77.6 | 13.5(10.0-436.9) | 0.0.6924 |

VITAMIN E SERUM

|  | Less than Reference  Range (n=22) | | Within range (n=365)  7.4~27.0 mg/L | | P  (P<0.05) | Greater than Reference  range | | Within range | | P  (P<0.05) |
| --- | --- | --- | --- | --- | --- | --- | --- | --- | --- | --- |
| T4 | 7.9 ± 1.7 | 7.5 (5.8-14) | 7.6 ± 1.6 | 7.4 (4.2-15.5) | 0.4233 | - | - | - | - | - |
| T3 | 1.2 ± 0.3 | 1.1 (0.8-2.2) | 1.1 ± 0.2 | 1.11 (0.5-3.0) | 0.1720 | - | - | - | - | - |
| TSH | 2.6 ± 1.4 | 2.6 (0.0-5.2) | 2.2 ± 1.8 | 1.8 (0.007-19.9) | 0.0806 | - | - | - | - | - |
| FT4 | 1.4 ± 0.2 | 1.39 (1.1-1.9) | 1.3 ± 0.2 | 1.2 (0.6-2.1) | 0.0763 | - | - | - | - | - |
| FT3 | 3.4 ± 0.8 | 3.2 (2.6-6.2) | 3.1 ± 0.5 | 3.09 (1.6-4.7) | 0.1474 | - | - | - | - | - |
| ATPO | 14.4 ± 6.3 | 13.1 (7.3-31.0) | 26.6 ± 55.2 | 10.7 (5-556.1) | 0.1894 | - | - | - | - | - |
| RT3 | 13.9 ± 4.2 | 14.0 (8.0-21.9) | 15.7 ± 24.2 | 13.4 (4.8-461) | 0.9865 | - | - | - | - | - |
| A-TG | 34.6 ± 88.0 | 13.3 (10.2-436.9) | 38.3 ± 75.6 | 13.5 (10.0-425) | 0.7005 | - | - | - | - | - |

CHOLINE SERUM

|  | Less than Reference  Range (n=21) | | Within range (n=365)  6.8~21.1 nmol/mL | | P  (P<0.05) | Greater than Reference  Range (n=1) | | Within range | | P  (P<0.05) |
| --- | --- | --- | --- | --- | --- | --- | --- | --- | --- | --- |
| T4 | 7.6 ± 1.6 | 7.5 (5.8-12.1) | 7.6 ± 1.6 | 7.45 (4.2-15.2) | 0.8071 | 7.0 | - | - | - | - |
| T3 | 1.1 ± 0.2 | 1.07 (0.7-1.5) | 1.1 ± 0.3 | 1.11 (0.5-3.0) | 0.7285 | 1.2 | - | - | - | - |
| TSH | 1.6 ± 0.7 | 1.64 (0.1-3.0) | 2.3 ± 1.8 | 1.85 (0.007-19.9) | 0.1119 | 1.9 | - | - | - | - |
| FT4 | 1.4 ± 0.2 | 1.3 (1.1-1.9) | 1.3 ± 0.2 | 1.3 (0.6-2.1) | 0.5001 | 1.4 | - | - | - | - |
| FT3 | 3.1 ± 0.5 | 3.12 (2.2-4.3) | 3.1 ± 0.5 | 3.09 (1.61-6.2) | 0.7994 | 3.9 | - | - | - | - |
| ATPO | 43.3 ± 73.3 | 11.3 (5.0-281.0) | 25.0 ± 52.2 | 10.7 (5.1-556.1) | 0.9137 | 8.1 | - | - | - | - |
| RT3 | 35.4 ± 95.2 | 13.4 (8.9-461.0) | 14.4 ± 6.3 | 13.4 (4.8-65.8) | 0.5898 | 18.2 | - | - | - | - |
| A-TG | 31.7 ± 43.3 | 12.5 (10.5-191.6) | 38.5 ± 77.9 | 13.5 (10-436.9) | 0.7076 | 10.9 | - | - | - | - |

INOSITOL SERUM

|  | Less than Reference  Range (n=9) | | Within range (n=373)  20.5~51.4 nmol/mL | | P  (P<0.05) | Greater than Reference  Range (n=5) | | Within range | | P  (P<0.05) |
| --- | --- | --- | --- | --- | --- | --- | --- | --- | --- | --- |
| T4 | 7.9 ± 1.3 | 8.3 (4.6-9.7) | 7.6 ± 1.6 | 7.4 (4.2-15.5) | 0.2147 | 8.0 ± 1.2 | 7.57 (6.4-9.8) | 7.6 ± 1.6 | 7.4 (4.2-15.5) | 0.0009 |
| T3 | 1.1 ± 0.2 | 1.07 (0.8-1.6) | 1.1 ± 0.3 | 1.1 (0.5-3.0) | 0.0535 | 1.2 ± 0.3 | 1.35 (0.6-1.5) | 1.1 ± 0.3 | 1.1 (0.5-3.0) | 0.0004 |
| TSH | 2.2 ± 1.2 | 1.6 (1.1-5.0) | 2.2 ± 1.8 | 1.8 (0.007-19.9) | 0.8071 | 2.5 ± 1.3 | 1.83 (1.6-5.1) | 2.2 ± 1.8 | 1.8 (0.007-19.9) | 0.5242 |
| FT4 | 1.3 ± 0.2 | 1.3 (0.9-1.5) | 1.3 ± 0.2 | 1.3 (0.63-2.1) | 0.8048 | 1.3 ± 0.3 | 1.39 (1.0-1.7) | 1.3 ± 0.2 | 1.3 (0.63-2.1) | 0.7865 |
| FT3 | 3.0 ± 0.3 | 2.9 (2.4-3.7) | 3.1 ± 0.5 | 3.1 (1.6-6.2) | 0.1776 | 3.1 ± 0.7 | 3.49 (1.9-3.8) | 3.1 ± 0.5 | 3.1 (1.6-6.2) | 0.6854 |
| ATPO | 31.7 ± 43.2 | 13.2 (6.7-147) | 25.5 ± 53.9 | 10.6 (5.0-556.1) | 0.2128 | 52.2 ± 49.2 | 18.3 (7.4-128.1) | 25.5 ± 53.9 | 10.6 (5.0-556.1) | 0.0980 |
| RT3 | 14.2 ± 4.3 | 13.4 (7.1-21.2) | 15.5 ± 23.8 | 13.4 (4.8-461) | 0.8380 | 27.1 ± 20.3 | 19.1 (7.5-65.8) | 15.5 ± 23.8 | 13.4 (4.8-461) | 0.1032 |
| A-TG | 52.2 ± 61.6 | 19.7 (12.6-210.3) | 36.7 ± 74.4 | 13.5 (10-436.9) | 0.0190 | 116.5 ± 160 | 13.6 (11-425.4) | 36.7 ± 74.4 | 13.5 (10-436.9) | 0.7727 |

CARNITINE SERUM

|  | Less than Reference  Range (n=8) | | Within range (n=375)  13.3~39.6 nmol/ml | | P  (P<0.05) | Greater than Reference  Range (n=4) | | Within range | | P  (P<0.05) |
| --- | --- | --- | --- | --- | --- | --- | --- | --- | --- | --- |
| T4 | 8.6 ± 2.7 | 7.81 (6.0-15) | 7.6 ± 1.5 | 7.4 (4.2-15.5) | 0.4101 | 7.1 ± 0.9 | 6.75 (6.4-8.7) | 7.6 ± 1.5 | 7.4 (4.2-15.5) | 0.0585 |
| T3 | 1.4 ± 0.7 | 1.20 (0.7-3.0) | 1.1 ± 0.2 | 1.11 (0.5-2.5) | 0.0310 | 0.9 ± 0.2 | 1.0 (0.6-1.1) | 1.1 ± 0.2 | 1.11 (0.5-2.5) | 0.1660 |
| TSH | 2.3 ± 1.5 | 1.8 ()0.6-5.4 | 2.2 ± 1.8 | 1.84 (0.007-19.9) | 0.9741 | 2.9 ± 1.6 | 2.6 (1.0-5.1) | 2.2 ± 1.8 | 1.84 (0.007-19.9) | 0.4535 |
| FT4 | 1.2 ± 0.1 | 1.2 (1.1-1.5) | 1.3 ± 0.2 | 1.3 (0.6-2.1) | 0.2286 | 1.3 ± 0.1 | 1.2 (1.1-1.4) | 1.3 ± 0.2 | 1.3 (0.6-2.1) | 0.6748 |
| FT3 | 3.2 ± 0.6 | 3 (2.2-4.3) | 3.1 ± 0.5 | 3.1 (1.61-6.2) | 0.8227 | 2.6 ± 0.5 | 2.8 (1.9-3.0) | 3.1 ± 0.5 | 3.1 (1.61-6.2) | 0.0569 |
| ATPO | 82.9 ± 72.4 | 51.2 (11.3-214.6) | 24.9 ± 52.8 | 10.6 (5-556.1) | 0.0002 | 10.2 ± 5 | 8.6 (5.4-18.3) | 24.9 ± 52.8 | 10.6 (5-556.1) | 0.3046 |
| RT3 | 68.8 ± 148.3 | 12.8 (9-461) | 14.3 ± 5.6 | 13.4 (4.8-39.6) | 0.9782 | 32.4 ± 19.6 | 23 (17.6-65.8) | 14.3 ± 5.6 | 13.4 (4.8-39.6) | 0.0029 |
| A-TG | 94.8 ± 129 | 32.2 (11.1-419.8) | 37 ± 74.8 | 13.5 (10-436) | 0.0050 | 13.1 ± 2.2 | 12 (11.3-16.9) | 37 ± 74.8 | 13.5 (10-436) | 0.3369 |

SODIUM SERUM

|  | Less than Reference  Range (n=11) | | Within range (n=333)  136~145 mmol/mL | | P  (P<0.05) | Greater than Reference  Range (n=43) | | Within range | | P  (P<0.05) |
| --- | --- | --- | --- | --- | --- | --- | --- | --- | --- | --- |
| T4 | 7.3 ± 1.1 | 7.5 (5.6-9.4) | 7.6 ± 1.6 | 7.3 (4.2-15.2) | 0.5201 | 7.7 ± 1.1 | 7.7 (5.3-10.5) | 7.6 ± 1.6 | 7.3 (4.2-15.2) | 0.3709 |
| T3 | 1.1 ± 0.3 | 1.0 (0.5-1.5) | 1.1 ± 0.3 | 1.1 (0.6-3.0) | 0.8014 | 1.1 ± 0.2 | 1.1 (0.7-1.6) | 1.1 ± 0.3 | 1.1 (0.6-3.0) | 0.3700 |
| TSH | 1.8 ± 0.5 | 1.8 (1.0-2.5) | 2.3 ± 1.9 | 1.85 (0.007-19.9) | 0.6269 | 2.1 ± 1.2 | 1.7 (0.0-5.3) | 2.3 ± 1.9 | 1.85 (0.007-19.9) | 0.7412 |
| FT4 | 1.3 ± 0.2 | 1.3 (1.0-1.6) | 1.3 ± 0.2 | 1.3 (0.6-2.0) | 0.6076 | 1.3 ± 0.2 | 1.3 (1.0-2.1) | 1.3 ± 0.2 | 1.3 (0.6-2.0) | 0.5219 |
| FT3 | 3.2 ± 0.7 | 3.0 (1.6-4.3) | 3.1 ± 0.5 | 3.1 (1.8-6.2) | 0.6774 | 3.1 ± 0.5 | 3 (2.2-4.4) | 3.1 ± 0.5 | 3.1 (1.8-6.2) | 0.4253 |
| ATPO | 46.4 ± 63.2 | 13 (5.6-214.6) | 26.1 ± 55.7 | 10.6 (5-556.1) | 0.1104 | 19.2 ± 26.6 | 10.8 (5.7-156.5) | 26.1 ± 55.7 | 10.6 (5-556.1) | 0.5090 |
| RT3 | 55 ± 128.4 | 15.5 (9.5-461) | 14.5 ± 6.4 | 13.4 (4.8-65.8) | 0.2514 | 13.8 ± 4.7 | 12.3 (5.7-25.1) | 14.5 ± 6.4 | 13.4 (4.8-65.8) | 0.8003 |
| A-TG | 26.8 ± 31.1 | 12.9 (10.2-121.2) | 35.8 ± 70.2 | 13.5 (10-436.9) | 0.9449 | 58.2 ± 116 | 14.4 (10.5-425) | 35.8 ± 70.2 | 13.5 (10-436.9) | 0.3701 |

/

POTASSIUM SERUM

|  | Less than Reference  Range (n=1) | | Within range | | P  (P<0.05) | Greater than Reference  Range (n=28) | | Within range (n=358)  3.5~5.1 mmol/L | | P  (P<0.05) |
| --- | --- | --- | --- | --- | --- | --- | --- | --- | --- | --- |
| T4 | 5.6 | - | - | - | - | 7.1 ± 1.4 | 7.4 (4.4-9.6) | 7.6 ± 1.6 | 7.4 (4.2-15.5) | 0.1372 |
| T3 | 1.1 | - | - | - | - | 1.1 ± 0.3 | 1.0 (0.7-2.2) | 1.1 ± 0.3 | 1.1 (0.5-3.0) | 0.1884 |
| TSH | 1.0 | - | - | - | - | 2.4 ± 2.1 | 2.0 (0.0-10.3) | 2.2 ± 1.7 | 1.8 (0.007-19.9) | 0.7563 |
| FT4 | 1.3 | - | - | - | - | 1.3 ± 0.2 | 1.2 (0.8-1.8) | 1.3 ± 0.2 | 1.3 (0.6-2.1) | 0.9846 |
| FT3 | 3.1 | - | - | - | - | 3.2 ± 0.7 | 3 (2.4-6.2) | 3.1 ± 0.5 | 3.1 (1.6-4.7) | 0.396 |
| ATPO | 8.1 | - | - | - | - | 31.4 ± 48.3 | 11.7 (5.4-210) | 25.5 ± 54.1 | 10.6 (5-556) | 0.1691 |
| RT3 | 9.5 | - | - | - | - | 13.8 ± 5.2 | 13.7 (6-25.1) | 15.7 ± 24.4 | 13.4 (4.8-461) | 0.8097 |
| A-TG | 12.3 | - | - | - | - | 52.3 ± 96.4 | 13.6 (10.2-420) | 36.9 ± 74.4 | 13.5 (10-436.9) | 0.9306 |

CALCIUM SERUM

|  | Less than Reference  Range (n=12) | | Within range (n=371)  8.9~10.6 mg/dL | | P  (P<0.05) | Greater than Reference  Range (n=4) | | Within range  8.9~10.6 mg/dL | | P  (P<0.05) |
| --- | --- | --- | --- | --- | --- | --- | --- | --- | --- | --- |
| T4 | 7.6 ± 1.1 | 7.3 (5.3-9.7) | 7.6 ± 1.6 | 7.4 (4.2-15.5) | 0.7165 | 7.7 ± 1.1 | 7.3 (5.3-9.7) | 7.6 ± 1.6 | 7.4 (4.2-15.5) | 0.6765 |
| T3 | 1.1 ± 0.2 | 1.1 (0.9-1.6) | 1.1 ± 0.3 | 1.1 (0.5-3.0) | 0.6883 | 1.1 ± 0.2 | 1.0 (0.9-1.5) | 1.1 ± 0.3 | 1.1 (0.5-3.0) | 0.7229 |
| TSH | 2.9 ± 1.5 | 2.4 (1.2-5.9) | 2.2 ± 1.8 | 1.8 (0.007-19.9) | 0.0594 | 2.1 ± 0.4 | 2.0 (1.6-2.6) | 2.2 ± 1.8 | 1.8 (0.007-19.9) | 0.5779 |
| FT4 | 1.2 ± 0.1 | 1.2 (1.0-1.4) | 1.3 ± 0.2 | 1.3 (0.6-2.1) | 0.2236 | 1.3 ± 0.1 | 1.2 (1.1-1.4) | 1.3 ± 0.2 | 1.3 (0.6-2.1) | 0.6376 |
| FT3 | 2.9 ± 0.2 | 2.92 (2.6-3.3) | 3.2 ± 0.5 | 3.1 (1.6-6.2) | 0.0266 | 2.8 ± 0.3 | 2.7 (2.4-3.1) | 3.2 ± 0.5 | 3.1 (1.6-6.2) | 0.0667 |
| ATPO | 43.7 ± 58.5 | 8.4 (6.6-182.6) | 25.5 ± 53.7 | 10.7 (5-556.1) | 0.7165 | 11.8 ± 3.1 | 13.1 (6.5-14.5) | 25.5 ± 53.7 | 10.7 (5-556.1) | 0.7599 |
| RT3 | 14.9 ± 4.8 | 13.7 (9.5-26.2) | 12.7 ± 24 | 13.5 (4.8-461) | 0.6381 | 10.8 ± 2.7 | 10.7 (7.1-14.6) | 12.7 ± 24 | 13.5 (4.8-461) | 0.1915 |
| A-TG | 68.3 ± 108 | 14.4 (10.9-372.2) | 37.4 ± 75.3 | 13.5 (10-436.9) | 0.3646 | 13.2 ± 3 | 13.0 (10.1-16.5) | 37.4 ± 75.3 | 13.5 (10-436.9) | 0.3696 |

MANGANESE SERUM

|  | Less than Reference  Range (n=2) | | Within range (n=381)  0.3~2.0 ng/mL | | P  (P<0.05) | Greater than Reference  Range (n=4) | | Within range  0.3~2.0 ng/mL | | P  (P<0.05) |
| --- | --- | --- | --- | --- | --- | --- | --- | --- | --- | --- |
| T4 | 8.7 ± 1.2 | 8.7 (7.5-9.9) | 7.6 ± 1.6 | 7.5 (4.2-15.5) | 0.2908 | 7.0 ± 1.1 | 6.6 (6.0-8.8) | 7.6 ± 1.6 | 7.5 (4.2-15.5) | 0.3948 |
| T3 | 1.4 ± 0.1 | 1.3 (1.2-1.5) | 1.1 ± 0.3 | 1.1 (0.5-3.0) | 0.1327 | 1.0 ± 0.2 | 0.9 (0.7-1.2) | 1.1 ± 0.3 | 1.1 (0.5-3.0) | 0.1043 |
| TSH | 1.2 ± 0.5 | 1.1 (0.7-1.7) | 2.2 ± 1.8 | 1.8 (0.007-19.9) | 0.2234 | 2.6 ± 1.8 | 1.9 (1.1-5.6) | 2.2 ± 1.8 | 1.8 (0.007-19.9) | 0.7038 |
| FT4 | 1.3 ± 0.0 | 1.25 (1.25-1.3) | 1.3 ± 0.2 | 1.3 (0.6-2.1) | 0.9429 | 1.2 ± 0.2 | 1.28 (0.9-1.4) | 1.3 ± 0.2 | 1.3 (0.6-2.1) | 0.5478 |
| FT3 | 3.0 ± 0.1 | 3.0 (2.9-3.2) | 3.1 ± 0.5 | 3.0 (1.6-6.2) | 0.8167 | 2.9 ± 0.6 | 2.6 (2.2-3.9) | 3.1 ± 0.5 | 3.0 (1.6-6.2) | 0.1768 |
| ATPO | 9.6 ± 3.0 | 9.5 (6.6-12.6) | 26.2 ± 54.1 | 10.8 (5-556.1) | 0.5200 | 9.7 ± 2.8 | 8.4 (7.4-14.4) | 26.2 ± 54.1 | 10.8 (5-556.1) | 0.2879 |
| RT3 | 19 ± 3.1 | 19 (15.9-22.1) | 15.6 ± 23.7 | 13.4 (4.8-461) | 0.1749 | 14 ± 2.6 | 13.1 (11.3-18.2) | 15.6 ± 23.7 | 13.4 (4.8-461) | 0.8975 |
| A-TG | 119.3 ± 98 | 119.3 (21.4-217.3) | 37.7 ± 76.3 | 13.5 (10-436.9) | 0.0540 | 36.3 ± 37.3 | 16.7 (10.9-100.8) | 37.7 ± 76.3 | 13.5 (10-436.9) | 0.4716 |

COPPER SERUM

|  | Less than Reference  Range (n=7) | | Within range (n=371)  0.7~1.5 mcg/mL | | P  (P<0.05) | Greater than Reference  Range (n=9) | | Within range  0.7~1.5 mcg/mL | | P  (P<0.05) |
| --- | --- | --- | --- | --- | --- | --- | --- | --- | --- | --- |
| T4 | 7.5 ± 1.3 | 7.2 (5.7-10.2) | 7.5 ± 1.5 | 7.4 (4.2-15.5) | 0.9109 | 10.3 ± 1.9 | 10.5 (7.3-14.2) | 7.5 ± 1.5 | 7.4 (4.2-15.5) | <0.0001 |
| T3 | 1.1 ± 0.1 | 1.06 (0.9-1.3) | 1.1 ± 0.2 | 1.1 (0.5-3.0) | 0.4881 | 1.5 ± 0.2 | 1.53 (1.1-1.8) | 1.1 ± 0.2 | 1.1 (0.5-3.0) | <0.0001 |
| TSH | 2.0 ± 0.9 | 1.7 (0.7-3.5) | 2.3 ± 1.8 | 1.8 (0.008-19.9) | 0.9299 | 1.6 ± 0.7 | 1.5 (0.0-2.9) | 2.3 ± 1.8 | 1.8 (0.008-19.9) | 0.2935 |
| FT4 | 1.4 ± 0.2 | 1.32 (1.1-1.8) | 1.3 ± 0.2 | 1.3 (0.6-2.1) | 0.3087 | 1.4 ± 0.3 | 1.4 (1.0-1.9) | 1.3 ± 0.2 | 1.3 (0.6-2.1) | 0.2687 |
| FT3 | 3.1 ± 0.4 | 2.9 (2.6-3.7) | 3.1 ± 0.5 | 3.0 (1.6-6.2) | 0.8839 | 3.4 ± 0.5 | 3.3 (2.6-4.1) | 3.1 ± 0.5 | 3.0 (1.6-6.2) | 0.1306 |
| ATPO | 12.7 ± 7 | 10.8 (5.4-28.3) | 26.4 ± 54.8 | 10.7 (5-556.1) | 0.8275 | 16.9 ± 12.5 | 12.3 (7.6-50.6) | 26.4 ± 54.8 | 10.7 (5-556.1) | 0.3465 |
| RT3 | 14.4 ± 5 | 13.8 (7.4-21.9) | 15.6 ± 23.9 | 13.4 (4.8-461) | 0.9262 | 17.0 ± 9.8 | 12.8 (7.5-36.5) | 15.6 ± 23.9 | 13.4 (4.8-461) | 0.9539 |
| A-TG | 14.3 ± 2.9 | 13.3 (11.2-19.8) | 38.5 ± 77.2 | 13.5 (10-436.9) | 0.7944 | 38.4 ± 70.6 | 12.7 (10.9-238.0) | 38.5 ± 77.2 | 13.5 (10-436.9) | 0.7631 |

CHROMIUM SERUM

|  | Less than Reference  Range (n=30) | | Within range (n=346)  0~0.7 ng/mL | | P  (P<0.05) | Greater than Reference  Range (n=11) | | Within range  0~0.7 ng/mL | | P  (P<0.05) |
| --- | --- | --- | --- | --- | --- | --- | --- | --- | --- | --- |
| T4 | 7.7 ± 1.8 | 7.5 (4.9-15) | 7.6 ± 1.6 | 7.4 (4.2-15.5) | 0.7521 | 7.5 ± 1.0 | 7.7 (6.3-8.8) | 7.6 ± 1.6 | 7.4 (4.2-15.5) | 0.9120 |
| T3 | 1.2 ± .04 | 1.1 (0.8-3.0) | 1.1 ± 0.2 | 1.1 (0.5-2.5) | 0.0955 | 1.1 ± 0.2 | 1.1 (0.8-1.6) | 1.1 ± 0.2 | 1.1 (0.5-2.5) | 0.7984 |
| TSH | 2.1 ± 1.1 | 2.0 (0.5-5.2) | 2.3 ± 1.8 | 1.8 (0.007-19.9) | 0.6799 | 2.2 ± 1.2 | 1.6 (1.2-5.6) | 2.3 ± 1.8 | 1.8 (0.007-19.9) | 0.9784 |
| FT4 | 1.2 ± 0.2 | 1.2 (0.9-1.6) | 1.3 ± 0.2 | 1.3 (0.6-2.1) | 0.0470 | 1.3 ± 0.1 | 1.3 (1.1-1.6) | 1.3 ± 0.2 | 1.3 (0.6-2.1) | 0.7680 |
| FT3 | 3.2 ± 0.4 | 3.1 (2.4-4.5) | 3.1 ± 0.5 | 3.0 (1.6-6.2) | 0.6570 | 3.0 ± 0.4 | 2.7 (2.5-3.8) | 3.1 ± 0.5 | 3.0 (1.6-6.2) | 0.2051 |
| ATPO | 21.2 ± 26 | 12.0 (5.3-128.1) | 26.9 ± 56.2 | 10.7 (5-556.1) | 0.4374 | 9.9 ± 2.4 | 9.4 (7.1-14.4) | 26.9 ± 56.2 | 10.7 (5-556.1) | 0.1748 |
| RT3 | 12.7 ± 4.2 | 12.4 (6-21.3) | 15.9 ± 24.8 | 13.6 (4.8-461) | 0.1069 | 12.5 ± 4.4 | 11.6 (7.1-22.5) | 15.9 ± 24.8 | 13.6 (4.8-461) | 0.2359 |
| A-TG | 31.3 ± 66.4 | 13.9 (10.3-372.2) | 39.4 ± 78.2 | 13.5 (10-436.9) | 0.9607 | 14.1 ± 2.4 | 13.8 (10.4-18.5) | 39.4 ± 78.2 | 13.5 (10-436.9) | 0.7490 |

IRON SERUM

|  | Less than Reference  Range (n=16) | | Within range (n=329)  35.0~150.0 mcg/dL | | P  (P<0.05) | Greater than Reference  Range (n=42) | | Within range | | P  (P<0.05) |
| --- | --- | --- | --- | --- | --- | --- | --- | --- | --- | --- |
| T4 | 7.4 ± 1.3 | 7.2 (5.0-9.7) | 7.6 ± 1.6 | 7.45 (4.2-15.5) | 0.7023 | 7.6 ± 1.5 | 7.46 (4.9-11.2) | 7.6 ± 1.6 | 7.45 (4.2-15.5) | 0.8925 |
| T3 | 1.1 ± 0.2 | 1.0 (0.6-1.3) | 1.1 ± 0.3 | 1.1 (0.5-3.0) | 0.3658 | 1.2 ± 0.3 | 1.1 (0.7-2.2) | 1.1 ± 0.3 | 1.1 (0.5-3.0) | 0.2909 |
| TSH | 2.9 ± 2.0 | 1.8 (0.8-7.8) | 2.3 ± 1.8 | 1.8 (0.007-19.9) | 0.4131 | 1.6 ± 1.8 | 1.7 (0.0-4.3) | 2.3 ± 1.8 | 1.8 (0.007-19.9) | 0.3971 |
| FT4 | 1.3 ± 0.2 | 1.2 (1.0-1.7) | 1.3 ± 0.2 | 1.2 (0.6-2.1) | 0.9954 | 1.3 ± 0.2 | 1.3 (0.9-1.7) | 1.3 ± 0.2 | 1.2 (0.6-2.1) | 0.1878 |
| FT3 | 2.9 ± 0.4 | 2.9 (1.9-3.3) | 3.1 ± 0.5 | 3.0 (1.6-4.7) | 0.0645 | 3.4 ± 0.6 | 3.25 (2.2-6.2) | 3.1 ± 0.5 | 3.0 (1.6-4.7) | 0.0045 |
| ATPO | 41.8 ± 68.0 | 10.4 (5.2-248) | 26.3 ± 55.7 | 10.8 (5.0-556) | 0.8913 | 17.1 ± 17.8 | 10.5 (6-79.0) | 26.3 ± 55.7 | 10.8 (5.0-556) | 0.9154 |
| RT3 | 18.8 ± 13.8 | 14.3 (7.3-65.8) | 15.6 ± 25.2 | 13.4 (4.8-461) | 0.3966 | 14.5 ± 5.4 | 13.3 (6.1-26.8) | 15.6 ± 25.2 | 13.4 (4.8-461) | 0.7872 |
| A-TG | 40 ± 67.7 | 14.6 (10.2-286.7) | 36.3 ± 80 | 13.5 (10-436.9) | 0.4189 | 27.7 ± 42.1 | 13.5 (10.2-238) | 36.3 ± 80 | 13.5 (10-436.9) | 0.8369 |

MAGNESIUM SERUM

|  | Less than Reference  Range (n=3) | | Within range (n=383)  1.6~2.6 mg/mL | | P  (P<0.05) | Greater than Reference  Range (n=1) | | Within range | | P  (P<0.05) |
| --- | --- | --- | --- | --- | --- | --- | --- | --- | --- | --- |
| T4 | 7.6 ± 1.4 | 6.7 (6.4-9.6) | 7.6 ± 1.6 | 7.4 (4.2-15.5) | 0.8748 | 11.0 | - | - | - | - |
| T3 | 1.1 ± 0.1 | 1.0 (1.0-1.2) | 1.1 ± 0.3 | 1.1 (0.5-3.0) | 0.9402 | 1.3 | - | - | - | - |
| TSH | 1.8 ± 0.6 | 1.4 (1.3-2.5) | 2.3 ± 1.8 | 1.8 (0.007-19.9) | 0.6588 | 1.3 | - | - | - | - |
| FT4 | 1.2 ± 0.2 | 1.1 (1.1-1.5) | 1.3 ± 0.2 | 1.3 (0.6-2.1) | 0.4574 | 1.4 | - | - | - | - |
| FT3 | 3.0 ± 0.1 | 2.8 (2.9-3.2) | 3.1 ± 0.5 | 3.0 (1.6-6.2) | 0.5084 | 2.9 | - | - | - | - |
| ATPO | 12.9 ± 7.0 | 9.9 (6.1-22.6) | 25.5 ± 52.7 | 10.7 (5.0-556.1) | 0.7381 | 252.4 | - | - | - | - |
| RT3 | 10.2 ± 4.9 | 9.0 (4.8-16.7) | 15.6 ± 23.6 | 13.4 (5.0-461.0) | 0.2305 | 19.1 | - | - | - | - |
| A-TG | 13.9 ± 2.4 | 13.5 (11.2-17.0) | 38 ± 76.6 | 13.5 (10-436.9) | 0.8134 | 136.2 | - | - | - | - |

VITAMIN A

|  | Less than Reference  Range (n=15) | | Within range (n=371)  40.8~150.3 mcg/dL | | P  (P<0.05) | Greater than Reference  Range (n=1) | | Within range | | P  (P<0.05) |
| --- | --- | --- | --- | --- | --- | --- | --- | --- | --- | --- |
| T4 | 7.4 ± 1.0 | 7.3 (5.7-9.7) | 7.6 ± 1.6 | 7.45 (4.2-15.5) | 0.6794 | - | - | - | - | - |
| T3 | 1.1 ± 0.2 | 1.0 (0.8-1.8) | 1.1 ± 0.3 | 1.1 (0.5-3.0) | 0.7831 | - | - | - | - | - |
| TSH | 2.7 ± 1.3 | 2.2 (1.0-5.2) | 2.2 ± 1.8 | 1.8 (0.007-19.9) | 0.1042 | - | - | - | - | - |
| FT4 | 1.3 ± 0.2 | 1.3 (0.9-1.6) | 1.3 ± 0.2 | 1.3 (0.6-2.1) | 0.8751 | - | - | - | - | - |
| FT3 | 3.2 ± 0.6 | 3.26 (2.4-4.5) | 3.1 ± 0.5 | 3.0 (1.6-6.2) | 0.7413 | - | - | - | - | - |
| ATPO | 51.6 ± 135.9 | 10.5 (5.3-556.1) | 25 ± 47.3 | 10.8 (5-324.5) | 0.8075 | - | - | - | - | - |
| RT3 | 13.9 ± 4.7 | 14.1 (7.7-24.6) | 15.7 ± 24 | 13.4 (4.5-461) | 0.8833 | - | - | - | - | - |
| A-TG | 33.1 ± 44.9 | 13.9 (10.3-157.8) | 38.3 ± 77.5 | 13.5 (10-436.9) | 0.7528 | - | - | - | - | - |

VITAMIN B1

|  | Less than Reference  Range (n=9) | | Within range (n=373)  1.40~71.3 nmol/L | | P  (P<0.05) | Greater than Reference  Range (n=5) | | Within range  1.40~71.3 nmol/L | | P  (P<0.05) |
| --- | --- | --- | --- | --- | --- | --- | --- | --- | --- | --- |
| T4 | 8.0 ± 2.7 | 7.1 (5.2-15.0) | 7.6 ± 1.5 | 7.4 (4.2-15.5) | 0.8071 | 6.7 ± 1.0 | 6.7 (5.3-8.1) | 7.6 ± 1.5 | 7.4 (4.2-15.5) | 0.1526 |
| T3 | 1.2 ± 0.7 | 0.99 (0.7-3.0) | 1.1 ± 0.2 | 1.2 (0.5-2.5) | 0.1761 | 1.0 ± 0.1 | 1.0 (0.9-1.1) | 1.1 ± 0.2 | 1.2 (0.5-2.5) | 0.1931 |
| TSH | 2.8 ± 3.3 | 1.8 (0.6-12.1) | 2.2 ± 1.7 | 1.8 (0.007-19.9) | 0.9252 | 3.0 ± 1.8 | 2.7 (1.3-6.5) | 2.2 ± 1.7 | 1.8 (0.007-19.9) | 0.2856 |
| FT4 | 1.3 ± 0.2 | 1.2 (1.0-1.5) | 1.3 ± 0.2 | 1.3 (0.6-2.1) | 0.4536 | 1.3 ± 0.2 | 1.2 (1.0-1.6) | 1.3 ± 0.2 | 1.3 (0.6-2.1) | 0.4905 |
| FT3 | 3.0 ± 0.4 | 2.9 (2.2-3.7) | 3.1 ± 0.5 | 3.1 (1.6-6.2) | 0.2727 | 3.1 ± 0.2 | 3.0 (2.9-3.3) | 3.1 ± 0.5 | 3.1 (1.6-6.2) | 0.8362 |
| ATPO | 42.2 ± 53.7 | 16.1 (8.8-181) | 25.7 ± 54 | 10.7 (5.0-556.1) | 0.0991 | 12.3 ± 5.3 | 10.8 (7.6-22.6) | 25.7 ± 54 | 10.7 (5.0-556.1) | 0.9389 |
| RT3 | 15.2 ± 5.6 | 14.3 (8.8-29.3) | 15.7 ± 23.9 | 13.4 (4.8-461) | 0.7135 | 11.1 ± 4.3 | 9.0 (6.0-16.2) | 15.7 ± 23.9 | 13.4 (4.8-461) | 0.2022 |
| A-TG | 50.8 ± 52.9 | 15.8 (11.6-157.8) | 38.1 ± 77.3 | 13.5 (10-436.9) | 0.1698 | 13.5 ± 2.3 | 12.9 (10.7-17) | 38.1 ± 77.3 | 13.5 (10-436.9) | 0.5348 |

VITAMIN_B2

|  | Less than Reference  Range (n=13) | | Within range (n=372)  5.6~126.1 mcg/L | | P  (P<0.05) | Greater than Reference  Range (n=2) | | Within range  5.6~126.1 mcg/L | | P  (P<0.05) |
| --- | --- | --- | --- | --- | --- | --- | --- | --- | --- | --- |
| T4 | 6.7 ± 0.9 | 6.5 (4.9-8.6) | 7.6 ± 1.6 | 7.4 (4.2-15.2) | 0.0097 | 8.3 ± 1.3 | 8.3 (7.1-9.6) | 7.6 ± 1.6 | 7.4 (4.2-15.2) | 0.4948 |
| T3 | 1.1 ± 0.1 | 1.1 (0.8-1.4) | 1.1 ± 0.3 | 1.1 (0.5-3.0) | 0.9985 | 1.3 ± 0.0 | 1.2 (1.3-1.3) | 1.1 ± 0.3 | 1.1 (0.5-3.0) | 0.1850 |
| TSH | 2.0 ± 0.6 | 1.9 (1.2-3.1) | 2.3 ± 1.8 | 1.8 (0.007-19.9) | 0.6980 | 2.6 ± 0.5 | 2.5 (2.1-3.1) | 2.3 ± 1.8 | 1.8 (0.007-19.9) | 0.3477 |
| FT4 | 1.3 ± 0.2 | 1.1 (1.0-1.7) | 1.3 ± 0.2 | 1.3 (0.6-2.1) | 0.1478 | 1.4 ± 0.1 | 1.3 (1.3-1.5) | 1.3 ± 0.2 | 1.3 (0.6-2.1) | 0.5662 |
| FT3 | 3.2 ± 0.5 | 3.2 (2.4-4.3) | 3.1 ± 0.5 | 3.0 (1.6-6.2) | 0.6813 | 3.4 ± 0.4 | 3.4 (3.1-3.8) | 3.1 ± 0.5 | 3.0 (1.6-6.2) | 0.3825 |
| ATPO | 32 ± 55.8 | 11.2 (5.3-214.6) | 25.7 ± 53.7 | 10.6 (5.0-556.1) | 0.6485 | 31.1 ± 19.8 | 31.0 (11.3-50.9) | 25.7 ± 53.7 | 10.6 (5.0-556.1) | 0.2807 |
| RT3 | 48.4 ± 119.2 | 14.2 (7.2-461.0) | 14.5 ± 6.3 | 13.4 (4.8-65.8) | 0.4639 | 11.7 ± 1.3 | 11.6 (10.4-13.0) | 14.5 ± 6.3 | 13.4 (4.8-65.8) | 0.5380 |
| A-TG | 17.9 ± 10.1 | 13.9 (10.3-42.0) | 38.9 ± 77.8 | 13.5 (10.0-436.9) | 0.9585 | 15.6 ± 4.5 | 15.5 (11.1-20) | 38.9 ± 77.8 | 13.5 (10.0-436.9) | 0.8524 |

VITAMIN_B3

|  | Less than Reference  Range | | Within range | | P  (P<0.05) | Greater than Reference  range (n=5) | | Within range (n=382)  2.6~36.1 ng/mL | | P  (P<0.05) |
| --- | --- | --- | --- | --- | --- | --- | --- | --- | --- | --- |
| T4 | - | - | - | - | - | 7.2 ± 0.8 | 7.3 (5.8-8.4) | 7.6 ± 1.6 | 7.4 (4.2-15.2) | 0.7142 |
| T3 | - | - | - | - | - | 1.1 ± 0.1 | 1.05 (1.0-1.3) | 1.1 ± 0.3 | 1.11 (0.5-3.0) | 0.9215 |
| TSH | - | - | - | - | - | 1.8 ± 0.5 | 1.67 (1.0-2.4) | 2.3 ± 1.8 | 1.84 (0.007-19.9) | 0.6997 |
| FT4 | - | - | - | - | - | 1.1 ± 0.2 | 1.25 (0.9-1.4) | 1.3 ± 0.2 | 1.3 (0.6-2.1) | 0.1443 |
| FT3 | - | - | - | - | - | 2.8 ± 0.2 | 2.8 (2.6-3.2) | 3.1 ± 0.5 | 3.0 (1.6-6.2) | 0.0846 |
| ATPO | - | - | - | - | - | 118.9 ± 218 | 10.9 (6.6-556.1) | 24.7 ± 46.7 | 10.7 (5-324.5) | 0.9680 |
| RT3 | - | - | - | - | - | 12.5 ± 2.3 | 12.4 (8.9-15.9) | 15.6 ± 23.6 | 13.5 (4.8-461) | 0.5586 |
| A-TG | - | - | - | - | - | 125.8 ± 113.1 | 38.8 (11.9-344.3) | 36.9 ± 74.7 | 13.5 (10-436.9) | 0.0497 |

VITAMIN_B6

|  | Less than Reference  Range (n=9) | | Within range (n=373) | | P  (P<0.05) | Greater than Reference  Range (n=5) | | Within range  2.8~76.2 ng/mL | | P  (P<0.05) |
| --- | --- | --- | --- | --- | --- | --- | --- | --- | --- | --- |
| T4 | 7.4 ± 1.2 | 7.15 (5.3-9.3) | 7.6 ± 1.6 | 7.4 (4.2-15.5) | 0.7805 | 6.0 ± 0.8 | 5.7 (5.3-7.7) | 7.6 ± 1.6 | 7.4 (4.2-15.5) | 0.0086 |
| T3 | 1.2 ± 0.2 | 1.2 (1.0-1.6) | 1.1 ± 0.3 | 1.1 (0.5-3.0) | 0.2436 | 1.0 ± 0.0 | 0.9 (0.9-1.0) | 1.1 ± 0.3 | 1.1 (0.5-3.0) | 0.0394 |
| TSH | 2.7 ± 1.3 | 2.1 (1.4-5.9) | 2.2 ± 1.8 | 1.8 (0.007-19.9) | 0.1465 | 4.9 ± 1.9 | 4.0 (2.7-7.8) | 2.2 ± 1.8 | 1.8 (0.007-19.9) | 0.0005 |
| FT4 | 1.3 ± 0.1 | 1.2 (1.0-1.5) | 1.3 ± 0.2 | 1.3 (0.6-2.1) | 0.3771 | 1.1 ± 0.2 | 1.13 (0.9-1.5) | 1.3 ± 0.2 | 1.3 (0.6-2.1) | 0.0490 |
| FT3 | 3.3 ± 0.5 | 3.3 (2.4-4.3) | 3.1 ± 0.5 | 3.0 (1.6-6.2) | 0.3412 | 3.0 ± 0.2 | 2.9 (2.9-3.4) | 3.1 ± 0.5 | 3.0 (1.6-6.2) | 0.5229 |
| ATPO | 35.5 ± 63.6 | 14 (5.6-214.6) | 25.9 ± 53.7 | 10.7 (5.0-556.1) | 0.4600 | 11.4 ± 3.7 | 10.5 (7.2-18.4) | 25.9 ± 53.7 | 10.7 (5.0-556.1) | 0.7821 |
| RT3 | 65.6 ± 139 | 14.3 (10.1-461) | 14.4 ± 6.2 | 13.4 (4.8-65.8) | 0.1623 | 11.3 ± 4.0 | 8.6 (7.7-16.9) | 14.4 ± 6.2 | 13.4 (4.8-65.8) | 0.2128 |
| A-TG | 41 ± 68 | 14.7 (10.4-231.8) | 38 ± 76.9 | 13.5 (10-436.9) | 0.6667 | 37.5 ± 48 | 12.9 (10.2-133.4) | 38 ± 76.9 | 13.5 (10-436.9) | 0.8393 |

VITAMIN_B12

|  | Less than Reference  Range (n=3) | | Within range  (232~1245 ng/L) | | P  (P<0.05) | Greater than Reference  Range (n=32) | | Within range (n=352)  232~1245 ng/mL | | P  (P<0.05) |
| --- | --- | --- | --- | --- | --- | --- | --- | --- | --- | --- |
| T4 | 8.3 ± 0.1 | 8.3 (8.2-8.4) | 7.6 ± 1.5 | 7.4 (4.2-15.5) | 0.1837 | 7.7 ± 1.8 | 7.5 (4.6-14) | 7.6 ± 1.5 | 7.4 (4.2-15.5) | 0.6575 |
| T3 | 1.1 ± 0.1 | 1.1 (1.0-1.3) | 1.1 ± 0.3 | 1.1 (0.5-3.0) | 0.9334 | 1.1 ± 0.2 | 1.0 (0.7-1.6) | 1.1 ± 0.3 | 1.1 (0.5-3.0) | 0.0773 |
| TSH | 2.2 ± 2.1 | 1.6 (0.0-5.0) | 2.2 ± 1.8 | 1.8 (0.007-19.9) | 0.8200 | 2.3 ± 1.9 | 1.8 (0.5-11.0) | 2.2 ± 1.8 | 1.8 (0.007-19.9) | 0.7742 |
| FT4 | 1.6 ± 0.1 | 1.54 (1.5-1.6) | 1.3 ± 0.2 | 1.3 (0.8-2.1) | 0.0187 | 1.4 ± 0.2 | 1.29 (0.6-1.9) | 1.3 ± 0.2 | 1.3 (0.8-2.1) | 0.2569 |
| FT3 | 3.3 ± 0.5 | 3.15 (2.9-4.0) | 3.2 ± 0.5 | 3.09 (1.6-6.2) | 0.5904 | 3.0 ± 05 | 3.0 (2.2-4.1) | 3.2 ± 0.5 | 3.09 (1.6-6.2) | 0.2683 |
| ATPO | 57.3 ± 70.1 | 8.8 (6.7-156.5) | 25.6 ± 53.7 | 10.7 (5.0-556.1) | 0.7627 | 27.3 ± 50.4 | 10.9 (6.3-281.0) | 25.6 ± 53.7 | 10.7 (5.0-556.1) | 0.3732 |
| RT3 | 16.4 ± 2.2 | 17.2 (13.4-18.5) | 15.6 ± 24.6 | 13.2 (4.8-461.0) | 0.2638 | 15.6 ± 6.3 | 14.6 (5.9-31.6) | 15.6 ± 24.6 | 13.2 (4.8-461.0) | 0.2034 |
| A-TG | 103 ± 126.9 | 13.8 (12.6-282.4) | 36.1 ± 73.5 | 13.5 (10.0-436.9) | 0.5078 | 53.1 ± 94.9 | 14.6 (10.4-419.8) | 36.1 ± 73.5 | 13.5 (10.0-436.9) | 0.5169 |

VITAMIN B5

|  | Less than Reference  Range (n=12) | | Within range | | P  (P<0.05) | Greater than Reference  Range (n=4) | | Within range (n=371)  22.7~429.2 mcg/L | | P  (P<0.05) |
| --- | --- | --- | --- | --- | --- | --- | --- | --- | --- | --- |
| T4 | 7.5 ± 1.2 | 7.6 (5.0-9.4) | 7.6 ± 1.6 | 7.4 (4.2-15.5) | 0.9400 | 6.2 ± 0.6 | 6.13 (5.3-7.1) | 7.6 ± 1.6 | 7.4 (4.2-15.5) | 0.0255 |
| T3 | 1.1 ± 0.1 | 1.1 (0.8-1.2) | 1.1 ± 0.3 | 1.1 (0.5-3.0) | 0.5498 | 1.0 ± 0.2 | 1.0 (0.6-1.3) | 1.1 ± 0.3 | 1.1 (0.5-3.0) | 0.4036 |
| TSH | 1.6 ± 0.5 | 1.5 (0.8-2.8) | 2.2 ± 1.8 | 1.8 (0.007-19.9) | 0.1769 | 4.1 ± 1.8 | 3.9 (2.1-6.5) | 2.2 ± 1.8 | 1.8 (0.007-19.9) | 0.0228 |
| FT4 | 1.3 ± 0.2 | 1.3 (1.0-1.7) | 1.3 ± 0.2 | 1.3 (0.63-2.1) | 0.9390 | 1.3 ± 0.2 | 1.3 (0.6-2.1) | 1.3 ± 0.2 | 1.3 (0.63-2.1) | 0.9065 |
| FT3 | 2.9 ± 0.3 | 2.8 (2.4-3.5) | 3.2 ± 0.5 | 3.1 (1.6-6.2) | 0.0571 | 3.0 ± 0.7 | 3.0 (1.9-3.8) | 3.2 ± 0.5 | 3.1 (1.6-6.2) | 0.8396 |
| ATPO | 10.8 ± 5.2 | 8.6 (5.3-25.6) | 26.6 ± 54.7 | 10.7 (5.0-556.1) | 0.1676 | 12.0 ± 3.9 | 11.0 (7.6-18.3) | 26.6 ± 54.7 | 10.7 (5.0-556.1) | 0.9227 |
| RT3 | 13.3 ± 4.0 | 13.6 (7.6-23.0) | 15.6 ± 23.8 | 13.4 (4.8-461.0) | 0.6250 | 25.1 ± 23.7 | 13.2 (7.9-65.8) | 15.6 ± 23.8 | 13.4 (4.8-461.0) | 0.9302 |
| A-TG | 13.1 ± 3.0 | 11.9 (10.3-19.8) | 39.2 ± 77.8 | 13.6 (10.0-436.9) | 0.0227 | 11.5 ± 0.9 | 11.2 (10.7-13.0) | 39.2 ± 77.8 | 13.6 (10.0-436.9) | 0.0237 |

VITAMIN C

|  | Less than Reference  Range (n=21) | | Within range | | P  (P<0.05) | Greater than Reference  Range (n=3) | | Within range (n=363)  0.2~1.1 mg/dL | | P  (P<0.05) |
| --- | --- | --- | --- | --- | --- | --- | --- | --- | --- | --- |
| T4 | 8.3 ± 2.1 | 8.1 (5.3-15.0) | 7.6 ± 1.5 | 7.4 (4.2-15.5) | 0.0923 | 6.7 ± 0.7 | 6.4 (6.1-7.6) | 7.6 ± 1.5 | 7.4 (4.2-15.5) | 0.2337 |
| T3 | 1.2 ± 0.5 | 1.0 (0.8-3.0) | 1.1 ± 0.2 | 1.11 (0.5-2.5) | 0.5997 | 1.1 ± 0.1 | 1.08 (1.1-1.3) | 1.1 ± 0.2 | 1.11 (0.5-2.5) | 0.7140 |
| TSH | 1.9 ± 1.4 | 1.6 (0.6-5.9) | 2.3 ± 1.8 | 1.8 (0.007-19.9) | 0.1008 | 3.7 ± 4.2 | 1.49 (0.0-9.6) | 2.3 ± 1.8 | 1.8 (0.007-19.9) | 0.7219 |
| FT4 | 1.4 ± 0.2 | 1.34 (1.0-1.9) | 1.3 ± 0.2 | 1.3 (0.6-2.1) | 0.0935 | 1.1 ± 0.0 | 1.15 (1.1-1.2) | 1.3 ± 0.2 | 1.3 (0.6-2.1) | 0.0506 |
| FT3 | 3.1 ± 0.5 | 3.0 (2.4-4.0) | 3.2 ± 0.1 | 3.17 (3.1-3.4) | 0.9948 | 3.2 ± 0.1 | 3.17 (3.1-3.4) | 3.2 ± 0.1 | 3.17 (3.1-3.4) | 0.5071 |
| ATPO | 18.5 ± 19.5 | 10.7 (5.1-84.7) | 26.5 ± 55.2 | 10.8 (5.0-556.1) | 0.8084 | 15.3 ± 7.4 | 10.2 (10-25.8) | 26.5 ± 55.2 | 10.8 (5.0-556.1) | 0.6662 |
| RT3 | 15.3 ± 6.3 | 14.3 (5.2-33.6) | 15.7 ± 24.2 | 13.4 (5.0-461.0) | 0.4543 | 9.2 ± 3.5 | 9.3 (4.8-13.5) | 15.7 ± 24.2 | 13.4 (5.0-461.0) | 0.1183 |
| A-TG | 32.1 ± 52.1 | 13.0 (10.3-238) | 38.6 ± 77.8 | 13.5 (10.0-436.9) | 0.5913 | 15.3 ± 3.7 | 13.5 (12.0-20.5) | 38.6 ± 77.8 | 13.5 (10.0-436.9) | 0.9189 |

VITAMIN D3

|  | Less than Reference  Range (n=4) | | Within range (n=383)  0.4~1.6 ng/mL | | P  (P<0.05) | Greater than Reference  range | | Within range | | P  (P<0.05) |
| --- | --- | --- | --- | --- | --- | --- | --- | --- | --- | --- |
| T4 | 8.9 ± 1.2 | 8.9 (7.4-10.4) | 7.6 ± 1.6 | 7.44 (4.2-15.5) | 0.0604 | - | - | - | - | - |
| T3 | 1.3 ± 0.3 | 1.23 (1.1-1.8) | 1.1 ± 0.3 | 1.1 (0.5-3.0) | 0.2003 | - | - | - | - | - |
| TSH | 3.1 ± 1.4 | 2.6 (1.7-5.2) | 2.2 ± 1.8 | 1.84 (0.007-19.9) | 0.1860 | - | - | - | - | - |
| FT4 | 1.4 ± 0.1 | 1.3 (1.3-1.6) | 1.3 ± 0.2 | 1.3 (0.6-2.1) | 0.3095 | - | - | - | - | - |
| FT3 | 3.5 ± 0.7 | 3.4 (2.7-4.5) | 3.1 ± 0.5 | 3.0 (1.6-6.2) | 0.2549 | - | - | - | - | - |
| ATPO | 10.9 ± 2.0 | 10.2 (8.9-14.2) | 26.1 ± 54 | 10.8 (5.0-556.1) | 0.8737 | - | - | - | - | - |
| RT3 | 12.4 ± 4.1 | 12.3 (8.0-16.9) | 15.6 ± 23.6 | 13.4 (4.8-461) | 0.5592 | - | - | - | - | - |
| A-TG | 14.9 ± 2.9 | 13.6 (12.3-19.8) | 38.3 ± 76.7 | 10.0 (10.0-436.9) | 0.8175 | - | - | - | - | - |

VITAMIN_K1

|  | Less than Reference  Range (n=1) | | Within range | | P  (P<0.05) | Greater than Reference  Range (n=3) | | Within range (n=383)  0~6.4 ng/mL | | P  (P<0.05) |
| --- | --- | --- | --- | --- | --- | --- | --- | --- | --- | --- |
| T4 | 6.1 | - | - | - | - | 6.7 ± 1.2 | 6.3 (5.3-8.3) | 7.6 ± 1.6 | 7.4 (4.2-15.5) | 0.2800 |
| T3 | 1.0 | - | - | - | - | 1.2 ± 0.2 | 1.1 (0.9-1.5) | 1.1 ± 0.3 | 1.1 (0.5-3.0) | 0.6824 |
| TSH | 4.9 | - | - | - | - | 3.8 ± 1.9 | 2.6 (2.3-6.5) | 2.2 ± 1.8 | 1.8 (0.007-19.9) | 0.0765 |
| FT4 | 1.2 | - | - | - | - | 1.1 ± 0.1 | 1.1 (1.0-1.2) | 1.3 ± 0.2 | 1.3 (0.6-2.1) | 0.0261 |
| FT3 | 3.1 | - | - | - | - | 3.0 ± 0.1 | 3.02 (2.9-3.0) | 3.1 ± 0.5 | 3.1 (1.6-6.2) | 0.5118 |
| ATPO | 78.7 | - | - | - | - | 11.2 ± 1.4 | 10.8 (9.6-13.0) | 25.9 ± 53.9 | 10.7 (5.0-556.1) | 0.9345 |
| RT3 | 12.0 | - | - | - | - | 11.8 ± 4.1 | 10.1 (7.9-17.5) | 15.6 ± 23.6 | 13.5 (4.8-461.0) | 0.4642 |
| A-TG | 235.9 | - | - | - | - | 12.4 ± 1.7 | 12.9 (10.1-14.0) | 37.8 ± 76.1 | 13.5 (10-436.9) | 0.3000 |

VITAMIN_K2

|  | Less than Reference  Range (n=1) | | Within range | | P  (P<0.05) | Greater than Reference  Range (n=3) | | Within range (n=383)  0~2.05 ng/mL | | P  (P<0.05) |
| --- | --- | --- | --- | --- | --- | --- | --- | --- | --- | --- |
| T4 | 7.5 | - | - | - | - | 7.8 ± 1.4 | 7.6 (6.1-9.5) | 7.6 ± 1.6 | 7.4 (4.2-15.5) | 0.8391 |
| T3 | 1.1 | - | - | - | - | 1.1 ± 0.2 | 1.2 (0.9-1.3) | 1.1 ± 0.3 | 1.1 (0.5-3.0) | 0.7305 |
| TSH | 2.7 | - | - | - | - | 2.8 ± 2.3 | 2.7 (0.0-5.5) | 2.2 ± 1.8 | 1.8 (0.007-19.9) | 0.6497 |
| FT4 | 1.3 | - | - | - | - | 1.3 ± 0.3 | 1.1 (1.1-1.7) | 1.3 ± 0.2 | 1.3 (0.6-2.1) | 0.8037 |
| FT3 | 3.1 | - | - | - | - | 3.2 ± 0.4 | 3.0 (2.9-3.7) | 3.1 ± 0.5 | 3.0 (1.6-6.2) | 0.7510 |
| ATPO | 11.8 | - | - | - | - | 45.5 ± 46.0 | 20.15 (6.3-110) | 25.8 ± 53.8 | 10.7 (5.0-556.1) | 0.5187 |
| RT3 | 9.0 | - | - | - | - | 12.5 ± 2.2 | 13.6 (9.3-14.4) | 15.6 ± 23.6 | 13.4 (4.8-461) | 0.6613 |
| A-TG | 11.8 | - | - | - | - | 176 ± 171.6 | 100.9 (14.2-413.7) | 37.1 ± 74.3 | 13.5 (10-436.9) | 0.0513 |

FOLATE_SERUM

|  | Less than Reference  Range (n=8) | | Within range (n=379)  >=1.5 ng/mL | | P  (P<0.05) | Greater than Reference  range | | Within range | | P  (P<0.05) |
| --- | --- | --- | --- | --- | --- | --- | --- | --- | --- | --- |
| T4 | 8.1 ± 1.7 | 7.81 (5.8-10.6) | 7.6 ± 1.6 | 7.45 (4.2-15.5) | 0.5028 | - | - | - | - | - |
| T3 | 1.2 ± 0.3 | 1.1 (0.9-1.8) | 1.1 ± 0.3 | 1.1 (0.5-3.0) | 0.6194 | - | - | - | - | - |
| TSH | 5.7 ± 5.7 | 3.7 (1.4-19.9) | 2.2 ± 1.5 | 1.8 (0.00-12.1) | 0.0071 | - | - | - | - | - |
| FT4 | 1.3 ± 0.2 | 1.2 (1.0-1.7) | 1.3 ± 0.2 | 1.3 (0.6-2.1) | 0.4957 | - | - | - | - | - |
| FT3 | 2.9 ± 0.2 | 2.8 (2.6-3.3) | 3.1 ± 0.5 | 3.1 (1.6-6.2) | 0.1180 | - | - | - | - | - |
| ATPO | 80.5 ± 101 | 20.4 (9.4-298.6) | 24.8 ± 51.6 | 10.7 (5.01-556.1) | 0.0125 | - | - | - | - | - |
| RT3 | 16.2 ± 4.1 | 15.2 (10.3-23.0) | 15.6 ± 23.7 | 13.4 (4.8-461) | 0.1850 | - | - | - | - | - |
| A-TG | 104.8 ± 133.5 | 35.6 (14.6-406.3) | 36.7 ± 74.1 | 13.47 (10-436.9) | 0.0003 | - | - | - | - | - |

VITAMIN_D__25

|  | Less than Reference  Range (n=81) | | Within range (n=296) | | P  (P<0.05) | Greater than Reference  Range (n=10) | | Within range  30.0~108.0 ng/mL | | P  (P<0.05) |
| --- | --- | --- | --- | --- | --- | --- | --- | --- | --- | --- |
| T4 | 7.8 ± 1.7 | 7.7 (4.7-15.2) | 7.5 ± 1.5 | 7.3 (4.2-15.0) | 0.1309 | 7.1 ± 0.6 | 7.2 (6.0-7.9) | 7.5 ± 1.5 | 7.3 (4.2-15.0) | 0.4376 |
| T3 | 1.2 ± 0.3 | 1.1 (0.8-2.5) | 1.1 ± 0.2 | 1.0 (0.5-3.0) | 0.0006 | 1.1 ± 0.2 | 1.0 (0.7-1.6) | 1.1 ± 0.2 | 1.0 (0.5-3.0) | 0.6114 |
| TSH | 2.4 ± 1.6 | 1.9 (0.3-7.8) | 2.2 ± 1.8 | 1.8 (0.007-19.9) | 0.4488 | 1.8 ± 0.7 | 1.8 (0.0-2.9) | 2.2 ± 1.8 | 1.8 (0.007-19.9) | 0.8770 |
| FT4 | 1.3 ± 0.2 | 1.3 (0.9-2.0) | 1.3 ± 0.2 | 1.2 (0.6-2.1) | 0.5213 | 1.3 ± 0.1 | 1.25 (1.2-1.6) | 1.3 ± 0.2 | 1.2 (0.6-2.1) | 0.4526 |
| FT3 | 3.3 ± 0.4 | 3.1 (2.4-4.6) | 3.1 ± 0.5 | 3.0 (2.3-3.9) | 0.0076 | 3.1 ± 0.4 | 3.1 (2.3-3.9) | 3.1 ± 0.5 | 3.0 (2.3-3.9) | 0.9936 |
| ATPO | 32.5 ± 61.7 | 10.6 (5.1-298.6) | 24.5 ± 52.0 | 10.8 (5.0-556.1) | 0.8101 | 14.1 ± 12.1 | 10 (7.3-49.6) | 24.5 ± 52.0 | 10.8 (5.0-556.1) | 0.5952 |
| RT3 | 15.2 ± 5.9 | 14.1 (5.2-31.6) | 15.7 ± 26.7 | 13.2 (4.8-461.0) | 0.1618 | 14.6 ± 5.8 | 12.9 (7.8-29.6) | 15.7 ± 26.7 | 13.2 (4.8-461.0) | 0.8902 |
| A-TG | 36.9 ± 74.9 | 13.0 (10.1-436.9) | 39.1 ± 77.9 | 13.6 (10-425.4) | 0.4382 | 17.6 ± 10.1 | 14.7 (11.5-46.8) | 39.1 ± 77.9 | 13.6 (10-425.4) | 0.8558 |

ISOLEUCINE

|  | Less than Reference  Range (n=1) | | Within range | | P  (P<0.05) | Greater than Reference  Range (n=2) | | Within range (n=384)  36.0~107.0 nmol/mL | | P  (P<0.05) |
| --- | --- | --- | --- | --- | --- | --- | --- | --- | --- | --- |
| T4 | 8.4 | - | - | - | - | 9.6 ± 2.5 | 9.6 (7.1-12.1) | 7.6 ± 1.6 | 7.4 (4.2-15.5) | 0.3777 |
| T3 | 1.0 | - | - | - | - | 1.1 ± 0.2 | 1.1 (1.0-1.3) | 1.1 ± 0.3 | 1.1 (0.5-3.0) | 0.9020 |
| TSH | 1.0 | - | - | - | - | 0.8 ± 0.6 | 0.7 (0.1-1.4) | 2.2 ± 1.8 | 1.8 (0.007-19.9) | 0.0966 |
| FT4 | 1.4 | - | - | - | - | 1.6 ± 0.3 | 1.5 (1.3-1.9) | 1.3 ± 0.2 | 1.3 (0.6-2.1) | 0.2119 |
| FT3 | 2.6 | - | - | - | - | 3.0 ± 0.2 | 2.9 (2.8-3.2) | 3.1 ± 0.5 | 3.0 (1.6-6.2) | 0.6831 |
| ATPO | 556.1 | - | - | - | - | 11.9 ± 0.1 | 11.9 (11.9-12.0) | 25.9 ± 53.7 | 10.7 (5.0-556.1) | 0.6569 |
| RT3 | 13.7 | - | - | - | - | 18.9 ± 6.9 | 18.8 (12-25.8) | 15.6 ± 23.5 | 13.4 (4.8-461.0) | 0.4616 |
| A-TG | 38.8 | - | - | - | - | 42.3 ± 29.7 | 42.2 (12.6-72.0) | 38.1 ± 76.4 | 13.5 (10-436.9) | 0.5932 |

VALINE

|  | Less than Reference  Range (n=10) | | Within range (n=367)  136.0~309.0 nmol/mL | | P  (P<0.05) | Greater than Reference  Range (n=10)  136.0~309.0 nmol/mL | | Within range  136.0~309.0 nmol/mL | | P  (P<0.05) |
| --- | --- | --- | --- | --- | --- | --- | --- | --- | --- | --- |
| T4 | 6.4 ± 1.2 | 6.7 (4.2-8.1) | 7.6 ± 1.5 | 7.4 (4.4-15.5) | 0.0156 | 8.1 ± 1.8 | 7.3 (6.3-12.1) | 7.6 ± 1.5 | 7.4 (4.4-15.5) | 0.5583 |
| T3 | 1.0 ± 0.2 | 1.0 (0.5-1.3) | 1.1 ± 0.3 | 1.1 (0.6-3.0) | 0.02233 | 1.1 ± 0.5 | 1.0 (0.7-1.5) | 1.1 ± 0.3 | 1.1 (0.6-3.0) | 0.4246 |
| TSH | 2.4 ± 2.7 | 1.6 (0.8-10.3) | 2.2 ± 1.8 | 2.1 (1.0-3.9) | 0.4315 | 2.1 ± 1.0 | 1.9 (0.1-3.9) | 2.2 ± 1.8 | 2.1 (1.0-3.9) | 0.7781 |
| FT4 | 1.1 ± 0.2 | 1.2 (0.8-1.3) | 1.3 ± 0.2 | 1.3 (0.6-2.1) | 0.0119 | 1.5 ± 0.2 | 1.3 (1.2-1.9) | 1.3 ± 0.2 | 1.3 (0.6-2.1) | 0.0483 |
| FT3 | 2.8 ± 0.5 | 2.9 (1.6-3.4) | 3.2 ± 0.5 | 3.1 (1.8-6.2) | 0.1409 | 3.0 ± 0.4 | 3.0 (2.2-3.6) | 3.2 ± 0.5 | 3.1 (1.8-6.2) | 0.5991 |
| ATPO | 23.7 ± 26.8 | 13.4 (6.0-93.9) | 26.4 ± 54.9 | 10.6 (5.0-556.1) | 0.4221 | 11.1 ± 1.2 | 10.9 (9.0-12.8) | 26.4 ± 54.9 | 10.6 (5.0-556.1) | 0.8523 |
| RT3 | 10.0 ± 3.1 | 8.8 (6.2-14.4) | 15.7 ± 24.1 | 13.6 (4.8-461.0) | 0.0065 | 15.4 ± 6.4 | 12.5 (9.3-28.4) | 15.7 ± 24.1 | 13.6 (4.8-461.0) | 0.8857 |
| A-TG | 29.2 ± 22.9 | 16.5 (11.4-79.5) | 37.7 ± 75.7 | 13.5 (10-436.9) | 0.1530 | 60 ± 121.2 | 13.3 (11.6-419.8) | 37.7 ± 75.7 | 13.5 (10-436.9) | 0.5524 |

LEUCINE

|  | Less than Reference  Range (n=13) | | Within range (n=373)  68.0~183.0 nmol/mL | | P  (P<0.05) | Greater than Reference  Range (n=1) | | Within range | | P  (P<0.05) |
| --- | --- | --- | --- | --- | --- | --- | --- | --- | --- | --- |
| T4 | 7.3 ± 1.2 | 7.0 (4.4-9.8) | 7.6 ± 1.6 | 7.4 (4.2-15.5) | 0.5910 | 12.1 | - | - | - | - |
| T3 | 1.0 ± 0.2 | 1.0 (0.5-1.3) | 1.1 ± 0.3 | 1.1 (0.6-3.0) | 0.1643 | 1.3 | - | - | - | - |
| TSH | 2.5 ± 2.5 | 1.4 (0.9-10.3) | 2.2 ± 1.7 | 1.85 (0.007-19.9) | 0.3931 | 0.1 | - | - | - | - |
| FT4 | 1.3 ± 0.2 | 1.3 (0.8-1.6) | 1.3 ± 0.2 | 1.3 (0.6-2.1) | 0.6287 | 1.9 | - | - | - | - |
| FT3 | 2.8 ± 0.5 | 2.8 (1.6-3.5) | 3.2 ± 0.5 | 3.1 (2.1-6.2) | 0.0177 | 3.2 | - | - | - | - |
| ATPO | 62.7 ± 144 | 12.7 (5.4-556.1) | 24.7 ± 47.1 | 10.7 (5.0-324.5) | 0.2973 | 12.0 | - | - | - | - |
| RT3 | 18.7 ± 14.4 | 14.3 (6.4-65.8) | 15.4 ± 23.8 | 13.3 (4.8-461.0) | 0.3344 | 25.8 | - | - | - | - |
| A-TG | 22.6 ± 15 | 16.0 (11.3-62.3) | 38.7 ± 77.7 | 13.5 (10.0-436.9) | 0.2711 | 12.6 | - | - | - | - |

CITRULLINE

|  | Less than Reference  Range (n=27) | | Within range (n=348)  17.0~46.0 nmol/mL | | P  (P<0.05) | Greater than Reference  Range (n=12) | | Within range | | P  (P<0.05) |
| --- | --- | --- | --- | --- | --- | --- | --- | --- | --- | --- |
| T4 | 8.4 ± 1.5 | 7.7 (6.0-11.5) | 7.6 ± 1.6 | 7.3 (4.2-15.5) | 0.0066 | 7.1 ± 1.4 | 7.3 (4.9-8.8) | 7.6 ± 1.6 | 7.3 (4.2-15.5) | 0.4031 |
| T3 | 1.2 ± 0.3 | 1.2 (0.8-1.8) | 1.1 ± 0.2 | 1.1 (0.5-3.0) | 0.0259 | 1.0 ± 0.2 | 1.0 (0.6-1.2) | 1.1 ± 0.2 | 1.1 (0.5-3.0) | 0.0304 |
| TSH | 2.0 ± 1.2 | 1.6 (0.7-5.6) | 2.3 ± 1.8 | 1.8 (0.007-19.9) | 0.2866 | 2.7 ± 1.8 | 1.7 (0.7-5.6) | 2.3 ± 1.8 | 1.8 (0.007-19.9) | 0.6340 |
| FT4 | 1.4 ± 0.2 | 1.3 (1.0-1.7) | 1.3 ± 0.2 | 1.29 (0.6-2.1) | 0.1487 | 1.3 ± 0.1 | 1.33 (1.0-1.6) | 1.3 ± 0.2 | 1.29 (0.6-2.1) | 0.7051 |
| FT3 | 3.3 ± 0.5 | 3.2 (2.4-4.5) | 3.1 ± 0.5 | 3.0 (1.6-6.2) | 0.1235 | 2.9 ± 0.4 | 2.9 (1.9-3.6) | 3.1 ± 0.5 | 3.0 (1.6-6.2) | 0.1390 |
| ATPO | 21.4 ± 30.2 | 11.9 (5.2-128.1) | 26.3 ± 55.5 | 10.6 (5.0-556.1) | 0.9711 | 24.4 ± 38.8 | 12.8 (6.6-152.1) | 26.3 ± 55.5 | 10.6 (5.0-556.1) | 0.3647 |
| RT3 | 16.1 ± 7.5 | 14.4 (7.5-36.2) | 15.4 ± 25.5 | 13.3 (4.8-461) | 0.3952 | 18.4 ± 15.0 | 15.6 (7.2-65.8) | 15.4 ± 25.5 | 13.3 (4.8-461) | 0.5041 |
| A-TG | 42.1 ± 84.5 | 14.7 (10.3-436.9) | 38.2 ± 76.7 | 13.5 (10-425.4) | 0.5601 | 24.8 ± 34.0 | 13.2 (11.3-136.7) | 38.2 ± 76.7 | 13.5 (10-425.4) | 0.7800 |

ARGININE

|  | Less than Reference  Range (n=34) | | Within range (n=350)  32.0~120.0 nmol/mL | | P  (P<0.05) | Greater than Reference  Range (n=3) | | Within range | | P  (P<0.05) |
| --- | --- | --- | --- | --- | --- | --- | --- | --- | --- | --- |
| T4 | 7.1 ± 1.2 | 7.0 (4.2-9.8) | 7.6 ± 1.6 | 7.4 (4.3-15.5) | 0.0385 | 8.9 ± 2.4 | 8.1 (6.4-12.1) | 7.6 ± 1.6 | 7.4 (4.3-15.5) | 0.5396 |
| T3 | 1.1 ± 0.3 | 1.0 (0.5-1.9) | 3.1 ± 0.5 | 3.0 (1.8-6.2) | <0.0001 | 1.3 ± 0.1 | 1.3 (1.2-1.4) | 3.1 ± 0.5 | 3.0 (1.8-6.2) | <0.0001 |
| TSH | 2.1 ± 1.2 | 2.0 (0.0-5.2) | 2.3 ± 1.8 | 1.8 (0.007-19.9) | 0.8401 | 1.2 ± 0.8 | 1.54 (0.1-1.9) | 2.3 ± 1.8 | 1.8 (0.007-19.9) | 0.2533 |
| FT4 | 1.3 ± 0.2 | 1.29 (0.8-1.7) | 1.3 ± 0.2 | 1.3 (0.6-2.1) | 0.3995 | 1.4 ± 0.3 | 1.18 (1.2-1.9) | 1.3 ± 0.2 | 1.3 (0.6-2.1) | 0.9028 |
| FT3 | 3.2 ± 0.6 | 3.0 (1.6-4.6) | 3.1 ± 0.5 | 3.0 (1.8-6.2) | 0.9759 | 3.3 ± 0.2 | 3.2 (3.1-3.5) | 3.1 ± 0.5 | 3.0 (1.8-6.2) | 0.4180 |
| ATPO | 33.8 ± 93.2 | 11.8 (5.6-556.1) | 25.3 ± 48.3 | 10.6 (5.0-324.5) | 0.0.5817 | 10.2 ± 2.5 | 11.9 (6.7-12.0) | 25.3 ± 48.3 | 10.6 (5.0-324.5) | 0.7003 |
| RT3 | 13.0 ± 4.6 | 11.6 (6.2-24.9) | 15.8 ± 24.6 | 13.5 (4.8-461.0) | 0.2013 | 20.9 ± 3.7 | 20.6 (16.9-25.8) | 15.8 ± 24.6 | 13.5 (4.8-461.0) | 0.0340 |
| A-TG | 25.8 ± 40.1 | 13.6 (10.2-235.9) | 39.5 ± 79.2 | 13.5 (10-436.9) | 0.7715 | 12.1 ± 0.6 | 12.4 (11.3-12.6) | 39.5 ± 79.2 | 13.5 (10-436.9) | 0.1820 |

Data are presented as the mean ± SD, median (min–max).

^a^ Mann-Whitney U Test.

**Table S2: Pearson r correlation of micronutrients with thyroid parameters**

|  | r | p |
| --- | --- | --- |
| ASPARAGINE | | |
| T4 | 0.00147 | 0.977 |
| FT4 | -0.0636 | 0.2118 |
| FT3 | -0.0219 | 0.667 |
| T3 | 0.0578 | 0.2566 |
| TSH | 0.1765 | 0.0005 |
| ATPO | -0.0538 | 0.2913 |
| RT3 | -0.0010 | 0.9843 |
| A-TG | -0.0249 | 0.6254 |
| GLUTAMINE | | |
| T4 | -0.1955 | 0.0001 |
| FT4 | -0.05599 | 0.2719 |
| FT3 | -0.01248 | 0.8066 |
| T3 | -0.1005 | 0.0482 |
| TSH | 0.03247 | 0.5243 |
| ATPO | -0.06714 | 0.1875 |
| RT3 | -0.0263 | 0.6059 |
| A-TG | -0.1363 | 0.0072 |
| SERINE | | |
| T4 | 0.06419 | 0.2077 |
| FT4 | -0.01958 | 0.701 |
| FT3 | -0.03857 | 0.4493 |
| T3 | 0.0462 | 0.3648 |
| TSH | 0.1186 | 0.0196 |
| ATPO | -0.03299 | 0.5176 |
| RT3 | -0.0002232 | 0.9965 |
| A-TG | 0.01152 | 0.8212 |
| COENZYME Q10 | | |
| T4 | -0.00053 | 0.9917 |
| FT4 | 0.01389 | 0.7854 |
| FT3 | -0.06678 | 0.1899 |
| T3 | -0.08203 | 0.1071 |
| TSH | 0.0144 | 0.7776 |
| ATPO | -0.04034 | 0.4288 |
| RT3 | -0.03186 | 0.5321 |
| A-TG | 0.05873 | 0.2491 |
| CYSTEINE | | |
| T4 | 0.01313 | 0.7968 |
| FT4 | 0.05733 | 0.2605 |
| FT3 | 0.008352 | 0.8699 |
| T3 | -0.01036 | 0.8391 |
| TSH | 0.04754 | 0.351 |
| ATPO | -0.00521 | 0.9187 |
| RT3 | -0.00211 | 0.9669 |
| A-TG | 0.06984 | 0.1703 |
| SELENIUM | | |
| T4 | 0.01351 | 0.7911 |
| FT4 | 0.115 | 0.0237 |
| FT3 | -0.01809 | 0.7228 |
| T3 | -0.09447 | 0.0634 |
| TSH | 0.007355 | 0.8853 |
| ATPO | 0.08655 | 0.0891 |
| RT3 | -0.00235 | 0.9632 |
| A-TG | 0.02949 | 0.563 |
| VITAMIN E | | |
| T4 | -0.06018 | 0.2376 |
| FT4 | -0.09522 | 0.0613 |
| FT3 | -0.1594 | 0.0017 |
| T3 | -0.1036 | 0.0417 |
| TSH | 0.03607 | 0.4793 |
| ATPO | -0.00403 | 0.9371 |
| RT3 | -0.02075 | 0.6841 |
| A-TG | 0.08144 | 0.1097 |
| CHOLINE | | |
| T4 | 0.008781 | 0.8633 |
| FT4 | -0.01207 | 0.8129 |
| FT3 | -0.01878 | 0.7126 |
| T3 | 0.002861 | 0.9553 |
| TSH | 0.1621 | 0.0014 |
| ATPO | -0.09248 | 0.0692 |
| RT3 | -0.08883 | 0.0809 |
| A-TG | -0.005677 | 0.9114 |
| INOSITOL | | |
| T4 | -0.04031 | 0.4291 |
| FT4 | -0.00952 | 0.852 |
| FT3 | -0.03315 | 0.5156 |
| T3 | -0.02993 | 0.5571 |
| TSH | 0.04702 | 0.3562 |
| ATPO | 0.02343 | 0.6458 |
| RT3 | 0.07787 | 0.1262 |
| A-TG | 0.02996 | 0.5568 |
| CARNITINE | | |
| T4 | -0.00873 | 0.8641 |
| FT4 | 0.1243 | 0.0144 |
| FT3 | -0.00241 | 0.9624 |
| T3 | -0.1065 | 0.0363 |
| TSH | -0.05969 | 0.2414 |
| ATPO | -0.05904 | 0.2465 |
| RT3 | -0.09454 | 0.0632 |
| A-TG | -0.0945 | 0.0633 |
| SODIUM | | |
| T4 | -0.03108 | 0.5421 |
| FT4 | 0.04595 | 0.3673 |
| FT3 | 0.02242 | 0.6602 |
| T3 | -0.05055 | 0.3213 |
| TSH | -0.0008173 | 0.9872 |
| ATPO | -0.1066 | 0.0361 |
| RT3 | -0.1341 | 0.0082 |
| A-TG | 0.04069 | 0.4247 |
| POTASSIUM | | |
| T4 | -0.07632 | 0.134 |
| FT4 | -0.04073 | 0.4242 |
| FT3 | 0.03237 | 0.5255 |
| T3 | -0.0274 | 0.591 |
| TSH | 0.02893 | 0.5705 |
| ATPO | -0.02131 | 0.6761 |
| RT3 | -0.08803 | 0.0837 |
| A-TG | 0.06387 | 0.21 |
| CALCIUM | | |
| T4 | 0.02603 | 0.6098 |
| FT4 | 0.135 | 0.0078 |
| FT3 | 0.1624 | 0.0013 |
| T3 | -0.00958 | 0.8511 |
| TSH | 0.000437 | 0.9932 |
| ATPO | -0.06232 | 0.2213 |
| RT3 | 0.06954 | 0.1722 |
| A-TG | -0.05904 | 0.2465 |
| MANGANESE | | |
| T4 | -0.05568 | 0.2746 |
| FT4 | -0.09603 | 0.0591 |
| FT3 | -0.04929 | 0.3334 |
| T3 | -0.05357 | 0.2931 |
| TSH | 0.03564 | 0.4845 |
| ATPO | 0.02569 | 0.6144 |
| RT3 | -0.01236 | 0.8084 |
| A-TG | 0.03126 | 0.5398 |
| ZINC | | |
| T4 | 0.02057 | 0.6866 |
| FT4 | 0.1511 | 0.0029 |
| FT3 | 0.1195 | 0.0187 |
| T3 | -0.08117 | 0.1109 |
| TSH | 0.04329 | 0.3958 |
| ATPO | -0.04527 | 0.3745 |
| RT3 | -0.0507 | 0.3199 |
| A-TG | 0.02047 | 0.6881 |
| COPPER | | |
| T4 | 0.3462 | <0.0001 |
| FT4 | 0.005191 | 0.9189 |
| FT3 | -0.00944 | 0.8531 |
| T3 | 0.2789 | <0.0001 |
| TSH | -0.03258 | 0.5228 |
| ATPO | -0.03716 | 0.4661 |
| RT3 | -0.00568 | 0.9113 |
| A-TG | 0.07197 | 0.1577 |
| CHROMIUM | | |
| T4 | 0.01234 | 0.8088 |
| FT4 | 0.01372 | 0.7879 |
| FT3 | -0.05673 | 0.2656 |
| T3 | -0.03689 | 0.4693 |
| TSH | 0.02929 | 0.5657 |
| ATPO | -0.03588 | 0.4815 |
| RT3 | 0.0318 | 0.5328 |
| A-TG | -0.02965 | 0.5608 |
| IRON | | |
| T4 | -0.02001 | 0.6947 |
| FT4 | 0.08145 | 0.1096 |
| FT3 | 0.1981 | <0.0001 |
| T3 | 0.0278 | 0.5856 |
| TSH | -0.1263 | 0.0129 |
| ATPO | -0.0154 | 0.7627 |
| RT3 | -0.02239 | 0.6606 |
| A-TG | -0.08451 | 0.0969 |
| MAGNESIUM | | |
| T4 | 0.04227 | 0.407 |
| FT4 | 0.05628 | 0.2694 |
| FT3 | -0.0097 | 0.8492 |
| T3 | -0.05496 | 0.2808 |
| TSH | 0.06594 | 0.1955 |
| ATPO | -0.0071 | 0.8894 |
| RT3 | -0.02398 | 0.6381 |
| A-TG | 0.04588 | 0.3681 |
| VITAMIN A | | |
| T4 | 0.03043 | 0.5507 |
| FT4 | -0.03114 | 0.5414 |
| FT3 | 0.05764 | 0.258 |
| T3 | 0.09337 | 0.0665 |
| TSH | -0.01515 | 0.7664 |
| ATPO | -0.01011 | 0.8429 |
| RT3 | -0.01749 | 0.7316 |
| A-TG | -0.01549 | 0.7613 |
| VITAMIN_B1 | | |
| T4 | -0.03727 | 0.4647 |
| FT4 | -0.01028 | 0.8403 |
| FT3 | 0.03311 | 0.5161 |
| T3 | 0.0177 | 0.7285 |
| TSH | 0.002307 | 0.9639 |
| ATPO | 0.02604 | 0.6095 |
| RT3 | -0.03988 | 0.434 |
| A-TG | -0.07413 | 0.1455 |
| VITAMIN_B2 | | |
| T4 | 0.02694 | 0.5972 |
| FT4 | 0.007327 | 0.8858 |
| FT3 | 0.05642 | 0.2682 |
| T3 | 0.04572 | 0.3698 |
| TSH | 0.008065 | 0.8743 |
| ATPO | -0.06136 | 0.2285 |
| RT3 | -0.03457 | 0.4977 |
| A-TG | -0.0296 | 0.5616 |
| VITAMIN_B3 | | |
| T4 | -0.01795 | 0.7249 |
| FT4 | -0.06742 | 0.1856 |
| FT3 | -0.03192 | 0.5312 |
| T3 | 0.00001 | 0.9997 |
| TSH | 0.003566 | 0.9442 |
| ATPO | 0.002898 | 0.9547 |
| RT3 | -0.06473 | 0.2038 |
| A-TG | 0.1235 | 0.015 |
| VITAMIN_B6 | | |
| T4 | -0.1161 | 0.0224 |
| FT4 | -0.0538 | 0.2911 |
| FT3 | 0.02252 | 0.6588 |
| T3 | -0.08036 | 0.1145 |
| TSH | 0.1424 | 0.005 |
| ATPO | 0.02068 | 0.6851 |
| RT3 | -0.06159 | 0.2267 |
| A-TG | 0.0984 | 0.0531 |
| VITAMIN_B12 | | |
| T4 | -0.03668 | 0.4718 |
| FT4 | 0.05061 | 0.3207 |
| FT3 | -0.03406 | 0.504 |
| T3 | -0.1142 | 0.0247 |
| TSH | -0.02611 | 0.6086 |
| ATPO | 0.0332 | 0.5149 |
| RT3 | 0.03943 | 0.4393 |
| A-TG | -0.02392 | 0.6389 |
| VITAMIN_B5 | | |
| T4 | -0.01729 | 0.7345 |
| FT4 | 0.03473 | 0.4957 |
| FT3 | 0.0734 | 0.1495 |
| T3 | 0.02675 | 0.5999 |
| TSH | -0.00549 | 0.9142 |
| ATPO | -0.00933 | 0.8548 |
| RT3 | -0.01132 | 0.8243 |
| A-TG | -0.01807 | 0.723 |
| VITAMIN_C | | |
| T4 | -0.08282 | 0.1038 |
| FT4 | -0.07316 | 0.1509 |
| FT3 | 0.001928 | 0.9698 |
| T3 | -0.04438 | 0.3839 |
| TSH | -0.05398 | 0.2895 |
| ATPO | 0.000438 | 0.9931 |
| RT3 | -0.049 | 0.3364 |
| A-TG | 0.02412 | 0.6361 |
| VITAMIN_D3 | | |
| T4 | -0.07512 | 0.1402 |
| FT4 | -0.0089 | 0.8615 |
| FT3 | -0.09106 | 0.0736 |
| T3 | -0.1249 | 0.014 |
| TSH | 0.03405 | 0.5042 |
| ATPO | -0.02021 | 0.6919 |
| RT3 | 0.06729 | 0.1865 |
| A-TG | 0.1055 | 0.038 |
| VITAMIN_K1 | | |
| T4 | -0.1287 | 0.0113 |
| FT4 | -0.1781 | 0.0004 |
| FT3 | -0.08178 | 0.1082 |
| T3 | -0.02654 | 0.6027 |
| TSH | 0.05696 | 0.2636 |
| ATPO | 0.03681 | 0.4702 |
| RT3 | -0.04939 | 0.3325 |
| A-TG | -0.00935 | 0.8545 |
| VITAMIN_K2 | | |
| T4 | 0.02757 | 0.5887 |
| FT4 | -0.01527 | 0.7646 |
| FT3 | 0.01467 | 0.7736 |
| T3 | 0.03247 | 0.5242 |
| TSH | -0.00903 | 0.8595 |
| ATPO | -0.02963 | 0.5612 |
| RT3 | -0.04196 | 0.4104 |
| A-TG | 0.1071 | 0.0351 |
| FOLATE | | |
| T4 | -0.04271 | 0.4021 |
| FT4 | -0.001601 | 0.975 |
| FT3 | 0.048 | 0.3464 |
| T3 | -0.006625 | 0.8966 |
| TSH | -0.07755 | 0.1278 |
| ATPO | -0.01056 | 0.836 |
| RT3 | 0.03339 | 0.5126 |
| A-TG | -0.09052 | 0.0753 |
| VITAMIN_D__25_OH | | |
| T4 | -0.1027 | 0.0435 |
| FT4 | -0.06896 | 0.1758 |
| FT3 | -0.1165 | 0.0218 |
| T3 | -0.1417 | 0.0052 |
| TSH | 0.01699 | 0.7391 |
| ATPO | 0.01434 | 0.7786 |
| RT3 | -0.01826 | 0.7203 |
| A-TG | -0.03878 | 0.4469 |
| ISOLEUCINE | | |
| T4 | -0.03804 | 0.4555 |
| FT4 | -0.0266 | 0.6019 |
| FT3 | 0.0683 | 0.18 |
| T3 | 0.03426 | 0.5016 |
| TSH | 0.03821 | 0.4535 |
| ATPO | -0.1101 | 0.0303 |
| RT3 | -0.05982 | 0.2404 |
| A-TG | -0.02355 | 0.6443 |
| VALINE | | |
| T4 | 0.1474 | 0.0037 |
| FT4 | 0.1327 | 0.009 |
| FT3 | 0.006915 | 0.8921 |
| T3 | 0.03691 | 0.4691 |
| TSH | -0.06049 | 0.2351 |
| ATPO | -0.00909 | 0.8585 |
| RT3 | 0.02205 | 0.6654 |
| A-TG | -0.01697 | 0.7393 |
| LEUCINE | | |
| T4 | 0.1474 | 0.0037 |
| FT4 | 0.1326 | 0.009 |
| FT3 | 0.006997 | 0.8909 |
| T3 | 0.03701 | 0.4679 |
| TSH | -0.06052 | 0.2349 |
| ATPO | -0.00908 | 0.8587 |
| RT3 | 0.02204 | 0.6656 |
| A-TG | -0.01697 | 0.7392 |
| CITRULLINE | | |
| T4 | -0.2274 | <0.0001 |
| FT4 | -0.1089 | 0.0321 |
| FT3 | -0.1303 | 0.0103 |
| T3 | -0.1858 | 0.0002 |
| TSH | 0.08737 | 0.0861 |
| ATPO | -0.06598 | 0.1953 |
| RT3 | -0.03236 | 0.5257 |
| A-TG | -0.08161 | 0.1089 |
| ARGININE | | |
| T4 | 0.1592 | 0.0017 |
| FT4 | 0.06436 | 0.2065 |
| FT3 | 0.007477 | 0.8834 |
| T3 | 0.09147 | 0.0723 |
| TSH | -0.04163 | 0.4141 |
| ATPO | -0.02072 | 0.6845 |
| RT3 | -0.03129 | 0.5394 |
| A-TG | 0.06803 | 0.1817 |
